# A DNA topoisomerase IB in Thaumarchaeota testifies for the presence of this enzyme in the last common ancestor of Archaea and Eucarya

**DOI:** 10.1186/1745-6150-3-54

**Published:** 2008-12-23

**Authors:** Céline Brochier-Armanet, Simonetta Gribaldo, Patrick Forterre

**Affiliations:** 1 Université de Provence, Aix-Marseille I, CNRS UPR9043, Laboratoire de Chimie Bactérienne, IFR88, Marseille, France; 2 Institut Pasteur, 25 rue du Docteur roux, 75015 Paris, France; 3 Univ Paris-sud, Institut de Génétique et Microbiologie, CNRS UMR8621, 91405 Orsay Cedex, France

## Abstract

DNA topoisomerase IB (TopoIB) was thought for a long time to be a eukaryotic specific enzyme. A shorter version was then found in viruses and later on in several bacteria, but not in archaea. Here, we show that a eukaryotic-like TopoIB is present in the recently sequenced genomes of two archaea of the newly proposed phylum Thaumarchaeota. Phylogenetic analyses suggest that a TopoIB was present in the last common ancestor of Archaea and Eucarya. This finding indicates that the last common ancestor of Archaea and Eucarya may have harboured a DNA genome.

This article was reviewed by Eugene Koonin and Anthony Poole

## Findings

DNA topoisomerases are ubiquitous enzymes that control DNA topology and solve topological conflicts arising during DNA replication, transcription, and recombination [1-3] (For a recent review on DNA topoisomerases see also [4]). Based on their mechanisms of action, DNA topoisomerases belong to two classes, type I (Topo I) and type II (Topo II): Topo I change the number of DNA topological links by introducing transient single-stranded breaks in the DNA molecule, whereas Topo II introduce transient double-stranded breaks. According to phylogenetic criteria, both Topo II and Topo I classes regroup several families of unrelated (i.e. non homologous) proteins: Topo IIA and IIB on one hand, and Topo IA (that also includes the so-called Topo III of eukaryotes and bacteria), IB and IC on the other hand [5,6]. This indicates that enzymes with either Topo I or Topo II activity originated multiple times independently in the course of evolution. For instance, Topo IIA and IIB share a homologous ATP binding subunit, but their DNA cleavage-religation subunits are non homologous and are structurally unrelated [2,7]. Regarding Topo I enzymes, Topo IA, which form a transient covalent link in 5' of the DNA break during the reaction of topoisomerization, share a Toprim domain with Topo II, some nucleases and primases [8], whereas Topo IB, which form a transient covalent link in 3' of the DNA break, are distantly related to tyrosine recombinases [2,9]. Although Topo IC forms a 3' DNA link similarly to Topo IB, it harbors a novel unique fold, and is unrelated to Topo IB and tyrosine recombinases [10]. The three different Topo I families show very distinctive distributions in the living world: Topo IA are present in currently available complete genomes of organisms from the three domains of life [6], whereas Topo IC appears so far specific to one particular species, the archaeon *Methanopyrus kandleri *[5]. Finally, Topo IB is present in eukaryotes, in poxviruses, in the mimivirus, and in some bacteria [6,10,11].

**Figure 1:**
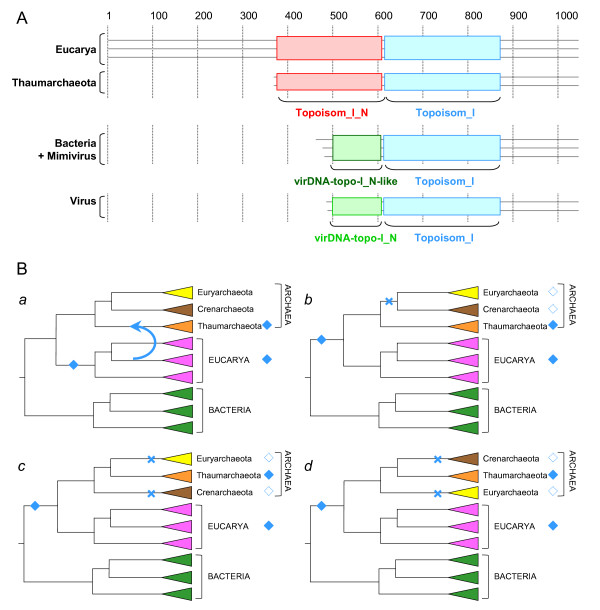
**A. Schematic representation of the domain organisation of Topo IB sequences from three eucarya, two thaumarchaeota, two bacteria and three viruses (the multiple alignment of these sequences is provided as Additional Files **1**)**. Coloured boxes delineate the putative functional domains according to the PFAM database : Topoisom_I_N (PF02919, Eukaryotic DNA topoisomerase I, DNA binding fragment) in red, Topoisom_I (PF01028, Eukaryotic DNA topoisomerase I, catalytic core) in blue and virDNA-Topo-I_N (PF09266, Viral DNA topoisomerase I, N-terminal) in light-green. The N-ter regions of viral Topo IB is similar in size and share conserved residues with bacterial and mimiviral homologues, suggesting the presence of a virDNA-Topo-I_N-like domain in these sequences (in dark-green). **B**. Alternative evolutionary scenarios explaining the presence of Topo IB in Thaumarchaeota. Filled blue diamonds indicate the presence of a Topo IB coding gene. Empty blue diamonds indicate a Topo IB coding gene that were present in the ancestor of the corresponding lineage and lost during its evolution. Blue crosses indicate the loss events of Topo IB coding genes. (**a**) A Topo IB coding gene was acquired by the ancestor of Thaumarchaeota via horizontal gene transfer (blue arrow) from a eukaryotic lineage. (**b**), (**c**) and (**d**) A Topo IB coding gene was present in the ancestor of Archaea and Eucarya and was subsequently lost in the ancestor of Crenarchaeota and Euryarchaeota, in agreement with a thaumarchaeal rooting of the archaeal tree (B). The Topo IB coding gene was independently lost in the ancestors of Euryarchaeota and Crenarchaeota according to an euryarchaeal or crenarchaeal rooting of the archaeal tree (C and D).

Topo IB (sometimes named swivelase) was first described in mouse and plays a very important role [1,12]. Indeed, whereas Topo IA can only relax negative superturns, Topo IB can relax both positive and negative superturns *in vitro*. As a consequence, eukaryotic Topo IB may relax the positive superturns that accumulate in front of replication forks or transcription bubbles during DNA replication, transcription, and chromatin assembly. In addition, Topo IB may also relax the compensatory positive superturns that form when the DNA becomes negatively wrapped around the histone octamer during nucleosome formation. Although these tasks can be fulfilled also by Topo II enzymes, genetic analyses have clearly indicated that Topo IB plays a major role in DNA replication, transcription and chromatin assembly in *Saccharomyces cerevisiae *[13-15]. Testifying for its crucial role in eukaryotes, Topo IB is the target of one of the most important antitumoral drugs, camptothecin [16]. Topo IB have been discovered in Poxviruses by Bauer and colleagues in 1977 [17], and the vaccinia virus Topo IB has been widely used as a model system to decipher the catalytic activity of this enzyme [18-20] and more recently to search for new antiviral drugs [21]. However, viral Topo IB are quite different from their eukaryotic counterparts, since they harbour a specific domain (virDNA-Topo-I_N) in their N-terminus instead of the long Topoisom_I_N domain found in eukaryotic homologues (Figure 1A and Additional files 1). Recently, homologues of Topo IB have been detected in several bacterial genomes and one of these has been characterized from *Deinococcus radiodurans *[22]. These bacterial Topo IB harbour a domain organisation close to the viral enzymes (Figure 1A and Additional files 1).

Up to now, Topo IB have never been observed in Archaea, in sharp contrast to members of the Topo IA family which are present in one or more copies in all archaeal genomes [6] (Additional files 2 and 3). Surprisingly, we recently noticed that a Topo IB coding gene was identified in the genome of the archaeon *Cenarchaeum symbiosum *[23,24], but that a Topo IA coding gene was absent [24]. Phylogenetic analyses of the archaeal domain based on concatenation of ribosomal proteins and comparative genome analysis have recently led us to propose that *C. symbiosum *and its relatives, formerly included in the phylum Crenarchaeota, should be considered as members of a separate and possibly ancient phylum, that we proposed to name Thaumarchaeota [24]. We predicted that the absence of a Topo IA and the presence of a Topo IB might be a distinctive feature of all thaumarchaeota members. As expected, we have detected an archaeal Topo IB homologue (YP_001582656), misannotated as an 2-alkenal reductase, in the recently sequenced genome of a second thaumarchaeon *Nitrosopumilus maritimus *[25], which also lacks a Topo IA homologue. Both thaumarchaeal Topo IB display a domain organisation that is very similar to that of their eukaryotic homologues, since these harbour both the N-terminal Topoisom_I_N and the C-terminal Topoisom_I domain (Figure 1A and Additional files 1). The main difference between the eukaryotic and the archaeal Topo IB is that the former possess a long and highly variable extension upstream of the Topoisom_I_N domain that is absent in the archaeal sequences (Figure 1A and Additional files 1). Two hypotheses can be proposed to explain the presence of a Topo IB coding gene in Thaumarchaeota. One is that this gene was acquired by the last common ancestor of Thaumarchaeota via a horizontal gene transfer (HGT) (blue arrow, Figure 1B-a). In that case, the donor would have been a eukaryote since both the thaumarchaeal and the eukaryotic Topo IB harbour a similar domain organisation. Alternatively, a Topo IB coding gene might have been present in the last common ancestor of Archaea and Eucarya and was then lost in all archaea, except in the lineage leading to Thaumarchaeota (Figures 1Bb-d). To distinguish between these two hypotheses on the origin of thaumarchaeal Topo IB, we have performed an in-depth phylogenetic analysis of Topo IB homologues.

**Figure 2:**
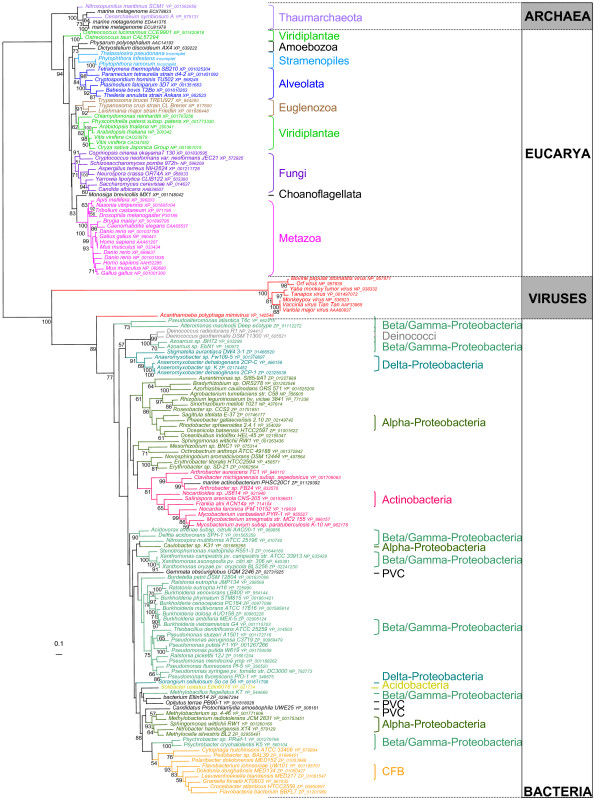
**Unrooted maximum likelihood phylogenetic tree of 151 Topo IB sequences**. Numbers at branches represent bootstrap proportions. The scale bar represents the average number of substitutions per site.

We retrieved homologues of Topo IB from the *nr *database at the NCBI (117 sequences from Eucarya, 2 from Archaea, 152 from Bacteria and 30 from viruses), as well as some environmental putative thaumarchaeal sequences from the GOS project [26] at the NCBI (For more details, see Additional files 2). We then selected 151 sequences representatives of Topo IB diversity for phylogenetic analysis. The resulting maximum likelihood tree (Figure 2) shows that the two archaeal Topo IB group with the few environmental sequences (BV = 100%) confirming that these are likely from yet uncultivated representatives of Thaumarchaeota. Although thaumarchaeal sequences are not yet abundant in environmental databases, this suggests that the presence of Topo IB is very likely a characteristic of this phylum. Moreover, thaumarchaeal Topo IB form a strongly supported sister-group to their eukaryotic homologues (BV = 100%). This sister-grouping of eukaryotic and thaumarchaeal sequences is also strongly supported when other reconstruction methods are used (not shown). The fact that thaumarchaeal sequences are sister to eukaryotes and do not arise from within them, coupled to the absence of the N-terminal extension in the archaeal sequences, strongly suggest that Thaumarchaeota did not acquire their Topo IB gene from a present-day eukaryotic lineage via a recent HGT. Based on phylogenetic and genomic analysis, we have recently proposed that Thaumarchaeota may represent the deepest branching lineage in the archaeal phylogeny, i.e. they emerged before the divergence between Euryarchaeota and Crenarchaeota [24]. This proposal is consistent with a large scale analysis performed by Koonin and collaborators [27]. The basal branching of Thaumarchaeota is also supported by the fact that, as in eukaryotes, the largest subunit of the RNA polymerase is not split in *C. symbiosum *and *N. maritimus *whereas it is split in A00 and A0 polypeptides in all other archaea for which sequences are available [28]. In order to account for the observed distribution of Topo IB in modern archaea, a deep branching of Thaumarchaeota requires only one evolutionary event (the loss of the Topo IB gene in an ancestor of Euryarchaeota and Crenarchaeota, after their divergence from Thaumarchaeota) (blue cross, Figure 1B-b). Accordingly, the presence of a Topo IB in *C. symbiosum *and *N. maritimus *may represent an ancestral archaeal feature. In contrast, if the root of the archaeal tree is located in either the euryarchaeal or the crenarchaeal branch, two independent losses of Topo IB would be required to explain the observed data (i.e. in the ancestor of Crenarchaeota and in the ancestor of Euryarchaeota, blue crosses, Figure 1B-c and 1B-d). Thus, the evolutionary history of Topo IB provides additional and independent evidence consistently with a rooting of the archaeal tree in the thaumarchaeal branch [24].

Topo IB have been for long thought to be absent in Archaea. Our finding now extends the presence of Topo IB homologues in members of all three domains of life. This may thus suggest that this enzyme was already present in the Last Universal Common Ancestor (LUCA). However, Topo IB homologues are either absent or scarcely distributed in complete genomes from most main bacterial phyla (Additional files 3). Moreover, the bacterial part of the Topo IB tree is not congruent with the bacterial specie tree (i.e. the monophyly of main bacterial groups is not recovered, Figure 2), suggesting that the history of Topo IB in Bacteria was dominated by lateral gene transfers. It was previously suggested that the viral-like Topo IB found in Bacteria was originally introduced from a DNA virus [6]. Our new and more detailed phylogenetic analysis, as well as the similarity of the domain organisation of viral and bacterial Topo IB, confirms the close relationship between these sequences and their probable common ancestry, although the direction of transfer is yet unclear.

The likely presence of both a Topo IA and Topo IB in the last common archaeal ancestor ([6] and this study, respectively), suggests that this ancestor was possibly more "complex" than modern archaea (if complexity is defined in terms of number of genes and/or redundancy of cellular processes). This idea was already proposed by Lecompte et *al. *who highlighted a streamlining in the evolution of archaeal ribosomes [29]. This is consistent with the recent observation that several proteins common to Archaea and Eukaryotes are missing in either Crenarchaeota, Euryarchaeota or Thaumarchaeota [27] and may indicate a possible tendency of evolution by streamlining of some central molecular processes in the archaeal domain. Finally, one of us has recently proposed that a transition from RNA genomes to DNA genomes occurred independently in each of the three life domains by the contribution of three different DNA viruses to three complex RNA cells [30]. The idea of different DNA viruses at the origin of Archaea and Eucarya sought to explain the existence of several critical differences in their DNA replication systems, including the ancestral presence of a Topo IB exclusively in Eucarya. Our finding that the last common ancestor of Archaea and Eucarya probably contained a Topo IB weakens this argument, and is more in favour of a DNA genome for this ancestor.

## Competing interests

The authors declare that they have no competing interests.

## Authors' contributions

CB, PF and SG conceived the study. CB designed and carried out the analyses, CB, SG and PF wrote the manuscript. All authors read and approved the final manuscript.

## Reviewers' comments

### Eugene Koonin

Review of Brochier-Armanet, gribaldo, and Forterre

'A DNA topoisomerase IB in Thaumarchaeota testifies for the presence of this enzyme in the last common ancestor of Archaea and Eukaryotes"

This is a very straightforward study of the Topo IB of Thaumarchaeota (formerly, mesophilic Crenarchaeota).demonstrating that the archaeal TopoIB clusters with the eukaryotic orthologs, at the base of the eukaryotic subtree. Combined with the fact that the archaeal and eukaryotic Topo IB proteins have similar domain organizations, these findings clearly demonstrate their monophyly.

1) I think, however, this is where the certainty stops. Indeed, I do not believe that the scenario with horizontal transfer of the eukaryotic Topo IB gene into the common ancestor of Thaumarchaeota can be considered rigorously falsified because it is hardly possible to rule out a dramatic acceleration of evolution after the transfer, resulting in the observed tree topology. PHyml is relatively robust to this sort of artifacts but there are inescapable limits. Ditto regarding the presence of Topo IB: the results of this work add credence to such a conclusion but alternatives based on horizontal gene transfer cannot be ruled out. I think the paper would become better balanced if these uncertainties were acknowledged, and the conclusions, especially, those at the end of the Abstract are toned down. In particular, the "support" of the Thaumaarcaheal rooting of the tree inferred from the phylogenetic analysis of this single gene is very weak, and it would be better to speak of the compatibility of the results with such rooting.

We think that the hypothesis of a HGT from present days eukaryotes to the ancestor of Thaumarchaeota is less likely than the hypothesis of the presence of a Topo IB gene in the ancestor of Eucarya and Archaea, followed by the loss of gene in the ancestor of Euryarchaeota/Crenarchaeota. However, as pointed out by referee two, we present both hypotheses and said carefully in the text that our phylogenetic analysis as the domain organisation of Topo IB homologues "strongly suggest".

Concerning the phylogenetic analyses, we used alternative methods to ML (as Bayesian methods), all the resulting trees strongly support the sister-grouping of Thaumarchaeota and Eucarya. We add this point in the text.

2) I also think that another adjustment, a less fundamental but, perhaps, even more badly needed one relates to the very "discovery" of the archaeal Topo IB. The protein sequence is very well conserved, so it is somewhat disingenuous to claim the finding of Topo IB as a discovery *sensu strictu*. The Cenarchaeum Topo IB is annotated in GenBank as such; it is another matter that the presence of this interesting gene in the *Cenarchaeum *genome is not highlighted in the primary paper (Hallam et al. PNAS 2006, 103: 18296) although "two topoisomerases" are mentioned. In any case, I do not think that it is proper to claim this finding in itself as a "discovery"; it would be much better to cite Hallam et al., and to explain the entire situation.

We cite the paper describing the genome of C. symbiosum and explain in the text, that one of the two DNA topoisomerases coding genes identified in the genome of C. symbiosum codes for a Topo IB, and that surprisingly no Topo IA coding gene was present in this genome.

Conversely, the ortholog from *Nitrosopumilis *is mistakenly annotated as some completely unrelated enzyme, and I think it is desirable to correct this (trivial) error. These corrections will not detract from the message of the present article but will make it better balanced.

We mention the fact that the gene coding for a putative homologue of TopoIB in N. maritimus was misannotated in this genome.

### Anthony Poole

This succinct report presents a nice phylogenetic result that provides two important evolutionary insights. The first is that the identification of Topo IB topoisomerases within members of the recently proposed archaeal phylum Thaumarchaeota (together with a supporting phylogenetic analysis) indicates that a Topo IB enzyme was likely present in the common ancestor of eukaryotes and archaea. This potentially tells us two things. First, if the presence of Topo IB within archaea is restricted to the Thaumarchaea, it strengthens the view that this is a genuine phylum (as recently proposed by these authors – ref. [24]). In that paper, the authors presented evidence that the mesophilic archaeon, *Crenarchaeum symbiosum *did not group within the Crenarchaea, and that, in their trees, this species was likewise distinct from Euryarchaeota. If the basal position of Thaumarchaeota is correct, the implication is that Topo IB was lost early in archaeal evolution, prior to the divergence of Euryarchaea and Crenarchaea. While their results (in ref. [24]) indicated that *C. symbiosum *is basal to the archaeal tree, in the current paper, they nevertheless approach this with caution, and provide us with three different scenarios (Figure 1B) that serve as a valuable framework for evaluating the implications of the conservation of eukaryotic and archaeal Topo IB (the fourth, transfer from eukaryotes – their Figure 1B-a – can be ruled out on the phylogenetic results presented). Figure 1B is therefore a very welcome addition to this paper because it allows the reader to evaluate the data and phylogeny in Figure 1B with respect to several hypotheses. Too often we see only one possible hypothesis being presented (and one sometimes gets a sense that the analysis of the data in a particular way is a foregone conclusion), so it is nice to see that the authors have thought this through carefully, and are both aware of and open to the compexities of interpretation.

The second insight is that placement of this topoisomerase type in the common ancestor of archaea and eukaryotes strengthens the evidence that this ancestor had a DNA-based genome. This point might need brief explanation. While the naïve expectation is that DNA was present in the Last Universal Common Ancestor, the available comparative genomic data on enzymes involved in deoxyribonucleotide synthesis and DNA replication do not allow this conclusion to be readily drawn. In light of these conflicting data, Forterre recently proposed a model (ref. [30]) wherein each domain may have independently gained the capacity for DNA synthesis. The essence of the model (an arms race between cells and viruses) is very elegant, and invokes known processes (there are several cases where viruses are known to carry altered genomes – phage genomes with uracil instead of thymine, for example). It is exciting to see that the discovery of Thaumarchaeal Topo IB helps to improve our understanding of DNA origins in that its inclusion supports a less complex scenario (i.e. at most two independent gains).

## Supplementary Material

Additional file 1 Archaeal-topoin-af1. Multiple alignment of Topo IB sequences from three eukaryotes (Scerevisiae = *Saccharomyces cerevisiae*, Hsapiens = *Homo sapiens*, Osativa = *Oryza sativa*), the two thaumarchaeota (*Cenarchaeum symbiosum *and *Nitrosopumilus maritimus*), two bacteria (Oterrae = Opitutus terrae PB90-1 and Rlitoralis = *Roseobacter litoralis Och 149*) and three viruses (Apolyphaga = Acanthamoeba polyphaga mimivirus, Bpapular = Bovine papular stomatitis virus and Ymonkey = Yaba monkey tumor virus). Coloured boxes delineate the putative functional domains according to the PFAM database: Topoisom_I_N (PF02919, Eukaryotic DNA topoisomerase I, DNA binding fragment) in red, Topoisom_I (PF01028, Eukaryotic DNA topoisomerase I, catalytic core) in blue and virDNA-Topo-I_N (PF09266, Viral DNA topoisomerase I, N-terminal) in green. The N-ter regions of viral Topo IB share conserved residues with bacterial and mimiviral homologues, suggesting the presence of a virDNA-Topo-I_N-like domain in these sequences. Click here for file

Additional file 2 Archaeal-topoin-af2. Material and methods. Click here for file

Additional file 3 Archaeal-topoin-af3. Table showing the taxonomic distribution of the 95 Topo IB, 634 Topo IA *sensu stricto*, 369 Topo III and 40 Reverse gyrase sequences retrieved from the 670 complete bacterial and archaeal genomes available in June 2008. Click here for file
