# Supplementary material for: A DNA topoisomerase IB in Thaumarchaeota testifies for the presence of this enzyme in the last common ancestor of Archaea and Eucarya
Source: Biol Direct. 2008 Dec 23;3:54. doi: 10.1186/1745-6150-3-54 (PMC2621148; doi:10.1186/1745-6150-3-54)
Supplement: Additional file 1 — Archaeal-topoin-af1. Multiple alignment of Topo IB sequences from three eukaryotes (Scerevisiae = Saccharomyces cerevisiae, Hsapiens = Homo sapiens, Osativa = Oryza sativa), the two thaumarchaeota (Cenarchaeum symbiosum and Nitrosopumilus maritimus), two bacteria (Oterrae = Opitutus terrae PB90-1 and Rlitoralis = Roseobacter litoralis Och 149) and three viruses (Apolyphaga = Acanthamoeba polyphaga mimivirus, Bpapular = Bovine papular stomatitis virus and Ymonkey = Yaba monkey tumor virus). Coloured boxes delineate the putative functional domains according to the PFAM database: Topoisom_I_N (PF02919, Eukaryotic DNA topoisomerase I, DNA binding fragment) in red, Topoisom_I (PF01028, Eukaryotic DNA topoisomerase I, catalytic core) in blue and virDNA-Topo-I_N (PF09266, Viral DNA topoisomerase I, N-terminal) in green. The N-ter regions of viral Topo IB share conserved residues with bacterial and mimiviral homologues, suggesting the presence of a virDNA-Topo-I_N-like domain in these sequences. [file 1745-6150-3-54-S1.pdf]

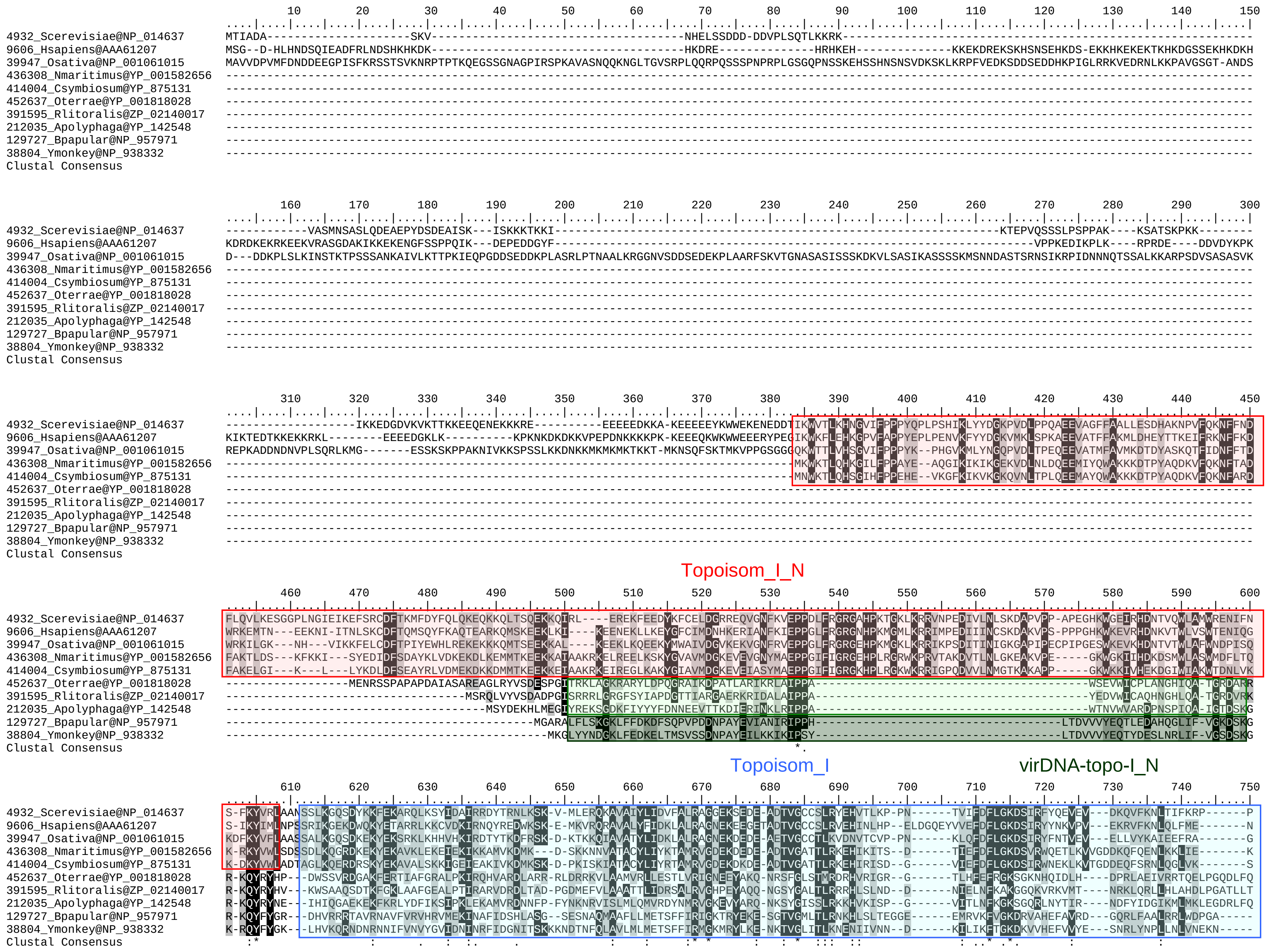

Topoisom_I_N
Topoisom_I
virDNA-topo-I_N
4932_Scerevisiae@NP_014637
9606_Hsapiens@AAA61207
39947_Osativa@NP_001061015
436308_Nmaritimus@YP_001582656
414004_Csymbiosum@YP_875131
452637_Oterrae@YP_001818028
391595_Rlitoralis@ZP_02140017
212035_Apolyphaga@YP_142548
129727_Bpapular@NP_957971
38804_Ymonkey@NP_938332
Clustal Consensus

```
                                        760       770       780       790       800       810       820       830       840       850       860       870       880       890       900
                                ....|....|....|....|....|....|....|....|....|....|....|....|....|....|....|....|....|....|....|....|....|....|....|....|....|....|....|....|....|....|
4932_Scerevisiae@NP_014637      KQ--PGHQLFD--RLDPSILNKYLQNYM--PGLTAKVFRTYNASKTMQDQLD---LIPN--KGSVAEKILKYNAANRTVAILCNHQRTVTKGHAQTVEKANNRIQELEWQKIRCKRAILQLDKDLLKKEPKYFEEIDDLTKEDEATIHKR
9606_Hsapiens@AAA61207          KQ--PEDDLFD--RLNTGILNKHLQDLM--EGLTAKVFRTYNASITLLKELT---APDEN-IPAKILSYNRANRAVAILCNHQRAPPKTFEKSMMNLQTKIDAKKEQL------------------------------------------
39947_Osativa@NP_001061015      KK--PGQDLFD--KLDTTRLNAHLKDLM--PGLTAKVFRTYNASITLDDILH---KETE--DGTLLEKIAVYQRANKEVAIICNHQRSVSKSHDSQMTRLNEKIDELKAQRDELKADLSKVRKGK-------------------------
436308_Nmaritimus@YP_001582656  KK--PSEEIFH--DITSRDVNKYLSGIV--KGVTAKVFRTYQATTVVKNYLV---NHDNIKGKSDNEKLYHAKLANLEAAIMCNHKRTIPKTFEQSLQKKKDTLKKREREK---------------------------------------
414004_Csymbiosum@YP_875131     IK--PKDEIFK--DIRSSDVNRYYSGIV--KGVTAKVFRTYLASKKVSEYLR---KNDKIRSKSAFFKLYHAKSANLEAAIMCNHKRTVPKTFKKALEKKKETLKKAKGAT---------------------------------------
452637_Oterrae@YP_001818028     YV--DDDGEPQ--KIGSADVNEYLREIAG-EEFSAKDFRTWAGTVLAALALR---ELGPFATKAEAKRNLVQAI--ERVSGRLGNTPAVCKKC---------------------------------------------------------
391595_Rlitoralis@ZP_02140017   WV--DDAGKPH--TLSSHQLNAYLKEAGGGAEITAKTFRTWAGTLAAF-LRA---EKGD--------ATIKDMA--TAASERLHNTPTVARNS---------------------------------------------------------
212035_Apolyphaga@YP_142548     YISTDEDGNEKIMRVNDRDLNKYIQENMG-SEFTIKDFRTFGANLYFIQALLSETRKRTPKNRKTIKKNIANAF--KSTARQLKHTGAVSKKS---------------------------------------------------------
129727_Bpapular@NP_957971       ----PEQLLFD--RLSERRVYAFMRRF----GIRVKDLRTYGVNYTFLYNFWSNV-RSLD-PRPSAKALICASV--RQTAETVGHTPSISRSA---------------------------------------------------------
38804_Ymonkey@NP_938332         ----PNGFLFN--KLSEKKVYEFMRKF----NIRIKDLRTYGVNYTFLYNFWSNV-KSIN-PLPNTKKLISMTI--KQTAEIVGHTPSISKNA---------------------------------------------------------
Clustal Consensus                         .   :    :  .         .  * :**:  .                                   .          .

                                        910       920       930       940       950       960       970       980       990       1000      1010      1020      1030      1040      1050
                                ....|....|....|....|....|....|....|....|....|....|....|....|....|....|....|....|....|....|....|....|....|....|....|....|....|....|....|....|....|....|
4932_Scerevisiae@NP_014637      IIDREIEKYQRKFVRENDKRKFE-KEELLPESQLKEWLEKVDEKKQEFEKELKTGEVELKSSWNSVEKIKAQVEKLEQRIQTSSIQLKDKEENSQVSLGTSKINYIDPRLSVVFCKKYDVPIEKIFTKT--LREK--FKWAIES---VDE
9606_Hsapiens@AAA61207          ----------------------------ADARRDLKSAKAD------AKVMKDAKTKK---VVESKKKAVQRLEEQLMKLEVQATDREENKQIALGTSKLNYLDPRITVAWCKKWGVPIEKIYNKT--QREK--FAWAIDM---ADEDYE
39947_Osativa@NP_001061015      -------------NLGNDKDGK-PKRNLA------------------------------------PEAFEKKISQIETKIEKMEMDKKIKEDLKTVALGTSKINYLDPRITVAWCKRHEVPIEKIFNKS--LIAK--FSWAMDV----DP
436308_Nmaritimus@YP_001582656  -----------------------------AWEKTQQTLKKVESSEPKTDTQKKNKEKRIKT---LNEQIKKQKQKHKERIEKLKLQVDLSEKTRDYNLGTSLRNYIDPRVFKAWTDEVGAEWEKLYTSA--LQKK--FLWVKNE----NT
414004_Csymbiosum@YP_875131     -----------------------------P----------------KTD---------------------KQKERKKERIEKIGLQIKLAQNTRDYNVGTSLRNYIDPRIFKAWTDEVGAEWEKLYTTA--LQRK--FLWVRDE----KE
452637_Oterrae@YP_001818028     --------------------------------------------------------------------------------------------------------YVHPVVLQSYLDGVTLEQVKAKAGAVLASAG--RNGNAGLSAEEKA
391595_Rlitoralis@ZP_02140017   --------------------------------------------------------------------------------------------------------YIHPAVIDLAG--QPAKSFDPI-------------KRNGLFAAEQR
212035_Apolyphaga@YP_142548     --------------------------------------------------------------------------------------------------------YVMNYTLELYQNNPEFFIEHKNDDPI------------------DF
129727_Bpapular@NP_957971       --------------------------------------------------------------------------------------------------------YMATAVLELVKDGTFLDRVAAT---------------DSLDDFVNS
38804_Ymonkey@NP_938332         --------------------------------------------------------------------------------------------------------YMANTVIDLLQKSDILKTIREI----------------DFNEFMNL
Clustal Consensus

                                        1060      1070      1080
                                ....|....|....|....|....|....|....
4932_Scerevisiae@NP_014637      NWR------------------------------F
9606_Hsapiens@AAA61207          --------------------------------F-
39947_Osativa@NP_001061015      DFR------------------------------F
436308_Nmaritimus@YP_001582656  KWKEIK----------------------------
414004_Csymbiosum@YP_875131     PWKKVSKQ-------------------------Y
452637_Oterrae@YP_001818028     VLAFLQRK-LPTTAERLAKSIRQEARKRGRGS-R
391595_Rlitoralis@ZP_02140017   LLGLLATQ--------------------------
212035_Apolyphaga@YP_142548     LLRILKSYRKDVLG-------------------E
129727_Bpapular@NP_957971       VVDYVNNS-EVVN--------------------G
38804_Ymonkey@NP_938332         VIGYVKNK-KII----------------------
Clustal Consensus
```
