# Supplementary material for: A DNA topoisomerase IB in Thaumarchaeota testifies for the presence of this enzyme in the last common ancestor of Archaea and Eucarya
Source: Biol Direct. 2008 Dec 23;3:54. doi: 10.1186/1745-6150-3-54 (PMC2621148; doi:10.1186/1745-6150-3-54)
Supplement: Additional file 2 — Archaeal-topoin-af2. Material and methods. [file 1745-6150-3-54-S2.pdf]

Material and methods

Homologues of Topo IB were retrieved from the *nr* and the environmental database (including sequences from the GOS project [1]) at the NCBI (http://www.ncbi.nlm.nih.gov/) using the BLASTp software [2]. For environmental sequences, only nearly complete sequences (i.e. longer than 350 amino acids positions) having an e-value lower than 10^{-30} using the sequence from *Cenarchaeum symbiosum* as seed were kept for phylogenetic analyses. Finally, 95 Topo IB and 1043 TopoIA homologues were retrieved from the 670 complete genomes of prokaryotes available in June 2008. The classification Topo IA sequences in TopoIA *sensu stricto*, Topo III and reverse gyrase was based on phylogenetic criteria.

Sequences were aligned by MUSCLE [3] and manually refined using *ed* from the MUST package [4]. Regions where homology was doubtful were manually removed from further analysis using the MUST package [4].

Maximum Likelihood (ML) phylogenetic trees were computed with PHYML [5] using the WAG model and a gamma correction to take into account the heterogeneity of evolutionary rates across sites (4 discrete classes of sites, an estimated alpha parameter and an estimated proportion of invariable sites). The robustness of each branch was estimated by the non-parametric bootstrap procedure implemented in PHYML (100 replicates of the original dataset and the same parameters) [5]. Additional phylogenetic analyses were performed using the bayesian method implemented in MrBayes [6] with a mixed model of amino acid substitution and a gamma correction (eight discrete categories plus a proportion of invariant sites) to take into account among-site rate variations. MrBayes was run with four chains for 1 million generations and trees were sampled every 100 generations. To construct the consensus tree, the first 1500 trees were discarded as ‘‘burnin.’’ a , and distance methods using.

To identify known functional domains, we performed searches in the Pfam database (http://www.sanger.ac.uk/Software/Pfam/). Alignments against the Pfam profiles having E-values lower than 0.1 were considered significant.

1. Venter JC, Remington K, Heidelberg JF, Halpern AL, Rusch D, Eisen JA, Wu D, Paulsen I, Nelson KE, Nelson W, et al: **Environmental genome shotgun sequencing of the Sargasso Sea.** *Science* 2004, **304:**66-74.
2. Altschul SF, Madden TL, Schaffer AA, Zhang J, Zhang Z, Miller W, Lipman DJ: **Gapped BLAST and PSI-BLAST: a new generation of protein database search programs.** *Nucleic Acids Res* 1997, **25:**3389-3402.

3. Edgar RC: **MUSCLE: a multiple sequence alignment method with reduced time and space complexity.** *BMC Bioinformatics* 2004, **5:**113.
4. Philippe H: **MUST, a computer package of Management Utilities for Sequences and Trees.** *Nucleic Acids Res* 1993, **21:**5264-5272.
5. Guindon S, Gascuel O: **A simple, fast, and accurate algorithm to estimate large phylogenies by maximum likelihood.** *Syst Biol* 2003, **52:**696-704.
6. Ronquist F, Huelsenbeck JP: **MrBayes 3: Bayesian phylogenetic inference under mixed models.** *Bioinformatics* 2003, **19:**1572-1574.
