# Supplementary material for: A DNA topoisomerase IB in Thaumarchaeota testifies for the presence of this enzyme in the last common ancestor of Archaea and Eucarya
Source: Biol Direct. 2008 Dec 23;3:54. doi: 10.1186/1745-6150-3-54 (PMC2621148; doi:10.1186/1745-6150-3-54)
Supplement: Additional file 3 — Archaeal-topoin-af3. Table showing the taxonomic distribution of the 95 Topo IB, 634 Topo IA sensu stricto, 369 Topo III and 40 Reverse gyrase sequences retrieved from the 670 complete bacterial and archaeal genomes available in June 2008. [file 1745-6150-3-54-S3.pdf]

| | Topoisomerase IB | Topoisomerase IA: Archaeal type / Bacterial type | Topoisomerase IA: Topoisomerase III | Topoisomerase IA: Reverse gyrase | Taxonomy |
|---|---|---|---|---|---|
| **ARCHAEA** | | | | | |
| **Crenarchaeota** | | | | | |
| *Aeropyrum pernix K1* | | NP_148170 | | NP_147880,NP_147906 | Desulfurococcales |
| *Caldivirga maquilingensis IC-167* | | YP_001539953 | | YP_001540636 | Thermoproteales |
| *Hyperthermus butylicus DSM 5456* | | YP_001013109 | | YP_001012435,YP_001013124 | Desulfurococcales |
| *Ignicoccus hospitalis KIN4/I* | | YP_001434598 | | YP_001435008,YP_001435105 | Desulfurococcales |
| *Metallosphaera sedula DSM 5348* | | | YP_001190937 | YP_001191827,YP_001192298 | Sulfolobales |
| *Pyrobaculum aerophilum str. IM2* | | NP_560414 | | NP_559079 | Thermoproteales |
| *Pyrobaculum arsenaticum DSM 13514* | | YP_001153574 | | YP_001152524 | Thermoproteales |
| *Pyrobaculum calidifontis JCM 11548* | | YP_001056006 | | YP_001055584 | Thermoproteales |
| *Pyrobaculum islandicum DSM 4184* | | YP_929913 | | YP_930753 | Thermoproteales |
| *Staphylothermus marinus F1* | | YP_001041270 | | YP_001040974,YP_001041125 | Desulfurococcales |
| *Sulfolobus acidocaldarius DSM 639* | | YP_255998 | | YP_255499 | Sulfolobales |
| *Sulfolobus solfataricus P2* | | NP_342400 | | NP_341957,NP_342447 | Sulfolobales |
| *Sulfolobus tokodaii str. 7* | | NP_377148 | | NP_376245,NP_377224 | Sulfolobales |
| *Thermofilum pendens Hrk 5* | | YP_920289 | | YP_919983 | Thermoproteales |
| *Thermoproteus neutrophilus V24Sta* | | YP_001795099 | | YP_001793605 | Thermoproteales |
| **Korarchaeota** | | | | | |
| *Korarchaeum termofilum OPF8* | | YP_001736678 | | YP_001737502 | |
| **Thaumarchaeota** | | | | | |
| *Nitrosopumilus maritimus SCM1* | YP_001582656 | | | | Nitrosopumilales |
| *Cenarchaeum symbiosum* | YP_875131 | | | | Cenarchaeales |
| **Euryarchaeota** | | | | | |
| *Archaeoglobus fulgidus DSM 4304* | | NP_070633 | | NP_069857 | Archaeoglobales |
| *Candidatus Methanoregula boonei 6A8* | | YP_001404020 | | | Methanomicrobiales |
| *Haloarcula marismortui ATCC 43049* | | YP_136265 | | | Halobacteriales |
| *Halobacterium sp. NRC-1* | | NP_444190 | | | Halobacteriales |
| *Halobacterium salinarum R1* | | YP_001688526 | | | Halobacteriales |
| *Haloquadratum walsbyi DSM 16790* | | YP_657047 | | | Halobacteriales |
| *Methanobrevibacter smithii ATCC 35061* | | YP_001273290 | | | Methanobacteriales |
| *Methanocaldococcus jannaschii DSM 2661* | | NP_248662 | | NP_248519 | Methanococcales |
| *Methanococcoides burtonii DSM 6242* | | YP_564864,YP_565420 | | | Methanosarcinales |
| *Methanococcus aeolicus Nankai-3* | | YP_001324231 | | | Methanococcales |
| *Methanococcus maripaludis C5* | | YP_001097306 | | | Methanococcales |
| *Methanococcus maripaludis C6* | | YP_001549743 | | | Methanococcales |
| *Methanococcus maripaludis C7* | | YP_001329424 | | | Methanococcales |
| *Methanococcus maripaludis S2* | | NP_988076 | | | Methanococcales |
| *Methanococcus vannielii SB* | | YP_001322681 | | | Methanococcales |
| *Methanocorpusculum labreanum Z* | | YP_001030575 | | | Methanomicrobiales |
| *Methanoculleus marisnigri JR1* | | YP_001046685 | | | Methanomicrobiales |
| *Methanopyrus kandleri AV19* | | NP_614887 | | NP_613576 | Methanopyrales |
| *Methanosaeta thermophila PT* | | YP_843026 | | | Methanosarcinales |
| *Methanosarcina acetivorans C2A* | | NP_616715,NP_617416 | | | Methanosarcinales |
| *Methanosarcina barkeri str. Fusaro* | | YP_305639,YP_305910 | | | Methanosarcinales |
| *Methanosarcina mazei Go1* | | NP_632207,NP_635100 | | | Methanosarcinales |
| *Methanosphaera stadtmanae DSM 3091* | | YP_447412 | | | Methanobacteriales |
| *Methanospirillum hungatei JF-1* | | YP_502480 | | | Methanomicrobiales |
| *Methanothermobacter thermautotrophicus str. Delta H* | | NP_276736 | | | Methanobacteriales |
| *Natronomonas pharaonis DSM 2160* | | YP_326271 | | | Halobacteriales |

| | | | | | |
|---|---|---|---|---|---|
| *Picrophilus torridus DSM 9790* | | YP_023525 | | | Thermoplasmatales |
| *Pyrococcus abyssi GE5* | | NP_127104 | | NP_126943 | Thermococcales |
| *Pyrococcus furiosus DSM 3638* | | NP_578223 | | NP_578224 | Thermococcales |
| *Pyrococcus horikoshii OT3* | | NP_142581 | | NP_142736 | Thermococcales |
| *Thermococcus kodakarensis KOD1* | | YP_183504 | | YP_182883 | Thermococcales |
| *Thermoplasma acidophilum DSM 1728* | | NP_393542 | | | Thermoplasmatales |
| *Thermoplasma volcanium GSS1* | | NP_110538 | | | Thermoplasmatales |
| *uncultured methanogenic archaeon RC-I* | | YP_686736,YP_686844 | | | |

## Nanoarchaeota

| | | | | | |
|---|---|---|---|---|---|
| *Nanoarchaeum equitans Kin4-M* | | NP_963339 | | NP_963605 | |

# BACTERIA

## Acidobacteria

| | | | | | |
|---|---|---|---|---|---|
| *Acidobacteria bacterium Ellin345* | | YP_592415 | | | Acidobacteriaceae |
| *Solibacter usitatus Ellin6076* | YP_821734 | YP_823673 | YP_828915 | | Solibacteraceae |

## Actinobacteria

| | | | | | |
|---|---|---|---|---|---|
| *Acidothermus cellulolyticus 11B* | | YP_873730 | | | Frankineae |
| *Arthrobacter aurescens TC1* | YP_946110 | YP_949030 | | | Micrococcineae |
| *Arthrobacter sp. FB24* | YP_832570 | YP_832830 | | | Micrococcineae |
| *Bifidobacterium adolescentis ATCC 15703* | | YP_909024 | | | Bifidobacteriaceae |
| *Bifidobacterium longum NCC2705* | | NP_695678 | NP_696628 | | Bifidobacteriaceae |
| *Clavibacter michiganensis subsp. michiganensis NCPPB 382* | YP_001223364 | YP_001221668 | | | Micrococcineae |
| *Clavibacter michiganensis subsp. Sepedonicus* | YP_001709063 | YP_001708973 | | | Micrococcineae |
| *Corynebacterium diphtheriae NCTC 13129* | | NP_938715 | | | Corynebacterineae |
| *Corynebacterium efficiens YS-314* | | NP_736917 | | | Corynebacterineae |
| *Corynebacterium glutamicum ATCC 13032* | | NP_599561 | | | Corynebacterineae |
| *Corynebacterium glutamicum ATCC 13032* | | YP_224609 | | | Corynebacterineae |
| *Corynebacterium glutamicum R* | | YP_001137261 | | | Corynebacterineae |
| *Corynebacterium jeikeium K411* | | YP_251753 | | | Corynebacterineae |
| *Corynebacterium urealyticum DSM 7109* | | YP_001801242 | | | Corynebacterineae |
| *Frankia alni ACN14a* | YP_714104 | YP_716706 | | | Frankineae |
| *Frankia sp. CcI3* | | YP_483380 | | | Frankineae |
| *Frankia sp. EAN1pec* | | YP_001504681 | | | Frankineae |
| *Kineococcus radiotolerans SRS30216* | | YP_001360241 | | | Frankineae |
| *Leifsonia xyli subsp. xyli str. CTCB07* | | YP_061514 | | | Micrococcineae |
| *Mycobacterium abscessus* | YP_001703237 | YP_001701242 | | | Corynebacterineae |
| *Mycobacterium avium 104* | | YP_879799 | | | Corynebacterineae |
| *Mycobacterium avium subsp. paratuberculosis K-10* | NP_962178 | NP_959359 | | | Corynebacterineae |
| *Mycobacterium bovis AF2122/97* | | NP_857309 | | | Corynebacterineae |
| *Mycobacterium bovis BCG str. Pasteur 1173P2* | | YP_979783 | | | Corynebacterineae |
| *Mycobacterium gilvum PYR-GCK* | YP_001133235 | YP_001132664 | | | Corynebacterineae |
| *Mycobacterium leprae TN* | | NP_301268 | | | Corynebacterineae |
| *Mycobacterium smegmatis str. MC2 155* | YP_886157 | YP_890377 | | | Corynebacterineae |
| *Mycobacterium sp. JLS* | YP_001069601 | YP_001073437 | | | Corynebacterineae |
| *Mycobacterium sp. KMS* | YP_937298 | YP_940862 | | | Corynebacterineae |
| *Mycobacterium sp. MCS* | YP_638447 | YP_641956 | | | Corynebacterineae |
| *Mycobacterium tuberculosis CDC1551* | | NP_338295 | | | Corynebacterineae |
| *Mycobacterium tuberculosis F11* | | YP_001289602 | | | Corynebacterineae |
| *Mycobacterium tuberculosis H37Ra* | | YP_001285032 | | | Corynebacterineae |
| *Mycobacterium tuberculosis H37Rv* | | NP_218163 | | | Corynebacterineae |
| *Mycobacterium ulcerans Agy99* | | YP_907724 | | | Corynebacterineae |
| *Mycobacterium vanbaalenii PYR-1* | YP_955527 | YP_956173 | | | Corynebacterineae |

| | | | | | |
|---|---|---|---|---|---|
| *Nocardia farcinica IFM 10152* | YP_119639 | YP_116573 | | | Corynebacterineae |
| *Nocardioides sp. JS614* | YP_921940 | YP_921608 | | | Propionibacterineae |
| *Propionibacterium acnes KPA171202* | | YP_054960 | | | Propionibacterineae |
| *Renibacterium salmoninarum ATCC 33209* | | YP_001625873 | | | Micrococcineae |
| *Rhodococcus sp. RHA1* | | YP_704301 | | | Corynebacterineae |
| *Rubrobacter xylanophilus DSM 9941* | | YP_644723 | YP_643532 | | Rubrobacterineae |
| *Saccharopolyspora erythraea NRRL 2338* | | YP_001102651 | | | Pseudonocardineae |
| *Salinispora arenicola CNS-205* | YP_001536631 | YP_001539176 | | | Micromonosporineae |
| *Salinispora tropica CNB-440* | YP_001158608 | YP_001160835 | | | Micromonosporineae |
| *Streptomyces avermitilis MA-4680* | | NP_825798 | | | Streptomycineae |
| *Streptomyces coelicolor A3(2)* | | NP_627741 | | | Streptomycineae |
| *Thermobifida fusca YX* | | YP_290840 | | | Streptosporangineae |
| *Tropheryma whipplei str. Twist* | | NP_787824 | | | Micrococcineae |
| *Tropheryma whipplei TW08/27* | | NP_789636 | | | Micrococcineae |
| **Aquificae** | | | | | |
| *Aquifex aeolicus VF5* | | NP_213453 | | NP_213599,NP_213790 | Aquificaceae |
| **Bacteroidetes/Chlorobi** | | | | | |
| *Bacteroides fragilis NCTC 9343* | | YP_213781 | YP_213185,YP_210918,YP_211372 | | Bacteroidales |
| *Bacteroides fragilis YCH46* | | YP_101691 | YP_101074,YP_098658,YP_097427 | | Bacteroidales |
| *Bacteroides thetaiotaomicron VPI-5482* | | NP_811739 | NP_811002,NP_809018,NP_811237,NP_811557 | | Bacteroidales |
| *Bacteroides vulgatus ATCC 8482* | | YP_001298646 | YP_001299249,YP_001298037,YP_001298832 | | Bacteroidales |
| *Candidatus Sulcia muelleri GWSS* | | | | | Flavobacteriales |
| *Chlorobium chlorochromatii CaD3* | | YP_378397 | | | Chlorobiales |
| *Chlorobium phaeobacteroides DSM 266* | | YP_910603 | | | Chlorobiales |
| *Chlorobium tepidum TLS* | | NP_660961 | | | Chlorobiales |
| *Cytophaga hutchinsonii ATCC 33406* | YP_678894 | YP_678943 | YP_680178 | | Sphingobacteriales |
| *Flavobacterium johnsoniae UW101* | YP_001195701 | YP_001194202 | YP_001194635,YP_001195361 | | Flavobacteriales |
| *Flavobacterium psychrophilum JIP02/86* | | YP_001296841 | | | Flavobacteriales |
| *Gramella forsetii KT0803* | YP_861935 | YP_863029 | | | Flavobacteriales |
| *Parabacteroides distasonis ATCC 8503* | | YP_001304919 | YP_001303061 | | Bacteroidales |
| *Pelodictyon luteolum DSM 273* | | YP_375958 | | | Chlorobiales |
| *Porphyromonas gingivalis W83* | | NP_905022 | NP_904451,NP_905641 | | Bacteroidales |
| *Prosthecochloris vibrioformis DSM 265* | | YP_001131226 | | | Chlorobiales |
| *Salinibacter ruber DSM 13855* | | YP_445245 | | | Sphingobacteriales |
| **PVC** | | | | | |
| *Candidatus Protochlamydia amoebophila UWE25* | YP_008181 | YP_007070 | | | Parachlamydiaceae |
| *Chlamydia muridarum Nigg* | | NP_296396 | | | Chlamydiaceae |
| *Chlamydia trachomatis 434/Bu* | | YP_001654102 | | | Chlamydiaceae |
| *Chlamydia trachomatis A/HAR-13* | | YP_328468 | | | Chlamydiaceae |
| *Chlamydia trachomatis D/UW-3/CX* | | NP_220161 | | | Chlamydiaceae |
| *Chlamydia trachomatis L2b/UCH-1/proctitis* | | YP_001653114 | | | Chlamydiaceae |
| *Chlamydophila abortus S26/3* | | YP_220342 | | | Chlamydiaceae |
| *Chlamydophila caviae GPIC* | | NP_829849 | | | Chlamydiaceae |
| *Chlamydophila felis Fe/C-56* | | YP_514942 | | | Chlamydiaceae |
| *Chlamydophila pneumoniae AR39* | | NP_445640 | | | Chlamydiaceae |
| *Chlamydophila pneumoniae CWL029* | | NP_224964 | | | Chlamydiaceae |
| *Chlamydophila pneumoniae J138* | | NP_300826 | | | Chlamydiaceae |
| *Chlamydophila pneumoniae TW-183* | | NP_877069 | | | Chlamydiaceae |
| *Rhodopirellula baltica SH 1* | | NP_868117 | | | Planctomycetales |
| **Chloroflexi** | | | | | |
| *Chloroflexus aurantiacus J-10-fl* | | YP_001637483 | | | Chloroflexaceae |
| *Dehalococcoides ethenogenes 195* | | YP_181458 | | | Dehalococcoides |

| | | | | | |
|---|---|---|---|---|---|
| *Dehalococcoides sp. BAV1* | | YP_001214115 | | | Dehalococcoides |
| *Dehalococcoides sp. CBDB1* | | YP_307755 | | | Dehalococcoides |
| *Herpetosiphon aurantiacus ATCC 23779* | | YP_001547149 | | | Herpetosiphonaceae |
| *Roseiflexus castenholzii DSM 13941* | | YP_001431800 | | | Chloroflexaceae |
| *Roseiflexus sp. RS-1* | | YP_001275778 | | | Chloroflexaceae |
| **Cyanobacteria** | | | | | |
| *Acaryochloris marina MBIC11017* | | YP_001516372,YP_001519161,YP_001521896 | | | unclassified |
| *Anabaena variabilis ATCC 29413* | | YP_320551,YP_324284 | | | Nostocaceae |
| *Cyanothece sp. ATCC 51142* | | YP_001802651,YP_001806222 | | | Chroococcales |
| *Gloeobacter violaceus PCC 7421* | | NP_923966,NP_926144 | | | Gloeobacterales |
| *Microcystis aeruginosa NIES-843* | | YP_001660677 | | | Microcystis |
| *Nostoc sp. PCC 7120* | | NP_486671,NP_486820 | | | Nostocaceae |
| *Prochlorococcus marinus str. AS9601* | | YP_001008886 | | | Prochlorococcaceae |
| *Prochlorococcus marinus str. MIT 9211* | | YP_001550318 | | | Prochlorococcaceae |
| *Prochlorococcus marinus str. MIT 9215* | | YP_001483717 | | | Prochlorococcaceae |
| *Prochlorococcus marinus str. MIT 9301* | | YP_001090684 | | | Prochlorococcaceae |
| *Prochlorococcus marinus str. MIT 9303* | | YP_001016647 | | | Prochlorococcaceae |
| *Prochlorococcus marinus str. MIT 9312* | | YP_396932 | | | Prochlorococcaceae |
| *Prochlorococcus marinus str. MIT 9313* | | NP_895180 | | | Prochlorococcaceae |
| *Prochlorococcus marinus str. MIT 9515* | | YP_001010814 | | | Prochlorococcaceae |
| *Prochlorococcus marinus str. NATL1A* | | YP_001014318 | | | Prochlorococcaceae |
| *Prochlorococcus marinus str. NATL2A* | | YP_292959 | | | Prochlorococcaceae |
| *Prochlorococcus marinus subsp. marinus str. CCMP1375* | | NP_874826 | | | Prochlorococcaceae |
| *Prochlorococcus marinus subsp. pastoris str. CCMP1986* | | NP_892554 | | | Prochlorococcaceae |
| *Synechococcus elongatus PCC 6301* | | YP_170849 | | | Chroococcales |
| *Synechococcus elongatus PCC 7942* | | YP_400433 | | | Chroococcales |
| *Synechococcus sp. CC9311* | | YP_731316 | | | Chroococcales |
| *Synechococcus sp. CC9605* | | YP_380926 | | | Chroococcales |
| *Synechococcus sp. CC9902* | | YP_377766 | | | Chroococcales |
| *Synechococcus sp. JA-2-3B'a(2-13)* | | YP_478560 | | | Chroococcales |
| *Synechococcus sp. JA-3-3Ab* | | YP_474040 | | | Chroococcales |
| *Synechococcus sp. PCC 7002* | | YP_001735778 | | | Chroococcales |
| *Synechococcus sp. RCC307* | | YP_001226916 | | | Chroococcales |
| *Synechococcus sp. WH 7803* | | YP_001225606 | | | Chroococcales |
| *Synechococcus sp. WH 8102* | | NP_897963 | | | Chroococcales |
| *Synechocystis sp. PCC 6803* | | NP_441184 | | | Chroococcales |
| *Thermosynechococcus elongatus BP-1* | | NP_682671 | | | Chroococcales |
| *Trichodesmium erythraeum IMS101* | | YP_720023 | | | Oscillatoriales |
| **Deinococcus-Thermus** | | | | | |
| *Deinococcus geothermalis DSM 11300* | YP_605521 | YP_605464 | | | Deinococcales |
| *Deinococcus radiodurans R1* | NP_294413 | NP_295097 | | | Deinococcales |
| *Thermus thermophilus HB27* | | YP_005900 | | | Thermales |
| *Thermus thermophilus HB8* | | YP_143340 | | YP_145411 | Thermales |
| **Firmicutes** | | | | | |
| *Alkaliphilus metalliredigens QYMF* | | YP_001320545 | YP_001321135,YP_001319011 | | Clostridiales |
| *Alkaliphilus oremlandii OhILAs* | | YP_001513014 | YP_001513687,YP_001512577 | | Clostridiales |
| *Acholeplasma laidlawii PG-8A* | | YP_001620337 | | | Mollicutes |
| *Aster yellows witches'-broom phytoplasma AYWB* | | YP_456638 | | | Mollicutes |
| *Bacillus amyloliquefaciens FZB42* | | YP_001421189 | YP_001420078 | | Bacillales |
| *Bacillus anthracis str. Ames* | | NP_846214 | NP_844316,NP_842919 | | Bacillales |
| *Bacillus anthracis str. 'Ames Ancestor'* | | YP_020610 | YP_018548,YP_016995,YP_022474 | | Bacillales |
| *Bacillus anthracis str. Sterne* | | YP_029935 | YP_028031,YP_026641 | | Bacillales |

| | | | | | |
|---|---|---|---|---|---|
| *Bacillus cereus ATCC 10987* | | NP_980172 | NP_978300,NP_976812,NP_982147 | | Bacillales |
| *Bacillus cereus ATCC 14579* | | NP_833552 | NP_831605,NP_830256 | | Bacillales |
| *Bacillus cereus E33L* | | YP_085174 | YP_083320,YP_081956 | | Bacillales |
| *Bacillus cereus subsp. cytotoxis NVH 391-98* | | YP_001375716 | YP_001374784,YP_001373711 | | Bacillales |
| *Bacillus clausii KSM-K16* | | YP_175774 | YP_177445,YP_175615 | | Bacillales |
| *Bacillus halodurans C-125* | | NP_243333 | NP_244253 | | Bacillales |
| *Bacillus licheniformis ATCC 14580* | | YP_079005 | YP_077752 | | Bacillales |
| *Bacillus licheniformis ATCC 14580* | | YP_091420 | YP_090167 | | Bacillales |
| *Bacillus pumilus SAFR-032* | | YP_001486753 | YP_001485655 | | Bacillales |
| *Bacillus subtilis subsp. subtilis str. 168* | | NP_389494 | NP_388307 | | Bacillales |
| *Bacillus thuringiensis serovar konkukian str. 97-27* | | YP_037894 | YP_036076,YP_034701,YP_173315,YP_035921 | | Bacillales |
| *Bacillus thuringiensis str. Al Hakam* | | YP_896206 | YP_894518,YP_893279 | | Bacillales |
| *Bacillus weihenstephanensis KBAB4* | | YP_001646455 | YP_001644629,YP_001643250,YP_001642732 | | Bacillales |
| *Caldicellulosiruptor saccharolyticus DSM 8903* | | YP_001180043 | | YP_001180363 | Clostridiales |
| *Carboxydothermus hydrogenoformans Z-2901* | | YP_360614 | | | Clostridiales |
| *Clostridium acetobutylicum ATCC 824* | | NP_348410 | NP_350150,NP_349549 | | Clostridiales |
| *Candidatus Desulforudis audaxviator MP104C* | | YP_001716766 | | | Clostridiales |
| *Clostridium beijerinckii NCIMB 8052* | | YP_001308324 | YP_001309000,YP_001308008 | | Clostridiales |
| *Clostridium botulinum A str. ATCC 19397* | | YP_001384612 | YP_001383644,YP_001383854 | | Clostridiales |
| *Clostridium botulinum A str. ATCC 3502* | | YP_001254936 | YP_001253811,YP_001254018 | | Clostridiales |
| *Clostridium botulinum A str. Hall* | | YP_001388129 | YP_001387193,YP_001387404 | | Clostridiales |
| *Clostridium botulinum A3 str. Loch Maree* | | YP_001787749 | YP_001786678,YP_001786927,YP_001715628 | | Clostridiales |
| *Clostridium botulinumB1 str. Okra* | | YP_001781980 | YP_001780912,YP_001781141 | | Clostridiales |
| *Clostridium botulinum F str. Langeland* | | YP_001391735 | YP_001390639,YP_001390851 | | Clostridiales |
| *Clostridium difficile 630* | | YP_001087768 | YP_001086889,YP_001088366,YP_001087593,YP_001088787,YP_001088916 | | Clostridiales |
| *Clostridium kluyveri DSM 555* | | YP_001394805 | YP_001394458,YP_001393961 | | Clostridiales |
| *Clostridium novyi NT* | | YP_878224 | YP_878833,YP_878362 | | Clostridiales |
| *Clostridium perfringens ATCC 13124* | | YP_696389 | YP_696196,YP_696662 | | Clostridiales |
| *Clostridium perfringens SM101* | | YP_698989 | YP_698808,YP_699260 | | Clostridiales |
| *Finegoldia magna ATCC 29328* | | YP_001692013 | YP_001692264,YP_001691185 | | Clostridiales |
| *Clostridium perfringens str. 13* | | NP_562618 | NP_562424,NP_562899,NP_150040 | | Clostridiales |
| *Clostridium phytofermentans ISDg* | | YP_001559822 | YP_001560365 | | Clostridiales |
| *Clostridium tetani E88* | | NP_781889 | NP_781100,NP_782419 | | Clostridiales |
| *Clostridium thermocellum ATCC 27405* | | YP_001036891 | | | Clostridiales |
| *Desulfitobacterium hafniense Y51* | | YP_518787 | YP_518305,YP_521140 | | Clostridiales |
| *Desulfotomaculum reducens MI-1* | | YP_001113327 | YP_001112334,YP_001113202,YP_001114550 | | Clostridiales |
| *Enterococcus faecalis V583* | | NP_815358 | NP_815968,NP_815727 | | Lactobacillales |
| *Exiguobacterium sibiricum 255-15* | | YP_001814357 | YP_001813458 | | Bacillales |
| *Geobacillus kaustophilus HTA426* | | YP_147064 | YP_147668,YP_147541 | | Bacillales |
| *Geobacillus thermodenitrificans NG80-2* | | YP_001125185 | YP_001125811,YP_001125646,YP_001127557 | | Bacillales |
| *Heliobacterium modesticaldum Ice1* | | YP_001680778 | YP_001680501 | | Clostridiales |
| *Lactobacillus acidophilus NCFM* | | YP_193863 | | | Lactobacillales |
| *Lactobacillus brevis ATCC 367* | | YP_794972 | | | Lactobacillales |
| *Lactobacillus casei ATCC 334* | | YP_806621 | | | Lactobacillales |
| *Lactobacillus delbrueckii subsp. bulgaricus ATCC 11842* | | YP_619140 | | | Lactobacillales |
| *Lactobacillus delbrueckii subsp. bulgaricus ATCC BAA-365* | | YP_813159 | YP_813304 | | Lactobacillales |
| *Lactobacillus gasseri ATCC 33323* | | YP_814743 | | | Lactobacillales |
| *Lactobacillus helveticus DPC 4571* | | YP_001577426 | | | Lactobacillales |
| *Lactobacillus johnsonii NCC 533* | | NP_964964 | | | Lactobacillales |
| *Lactobacillus plantarum WCFS1* | | NP_785400 | YP_133736 | | Lactobacillales |
| *Lactobacillus reuteri F275* | | YP_001271380 | | | Lactobacillales |
| *Lactobacillus sakei subsp. sakei 23K* | | YP_395600 | YP_395375 | | Lactobacillales |

| | | | | | |
|---|---|---|---|---|---|
| *Lactobacillus salivarius UCC118* | | YP_535613 | YP_536196,YP_536712 | | Lactobacillales |
| *Lactococcus lactis subsp. cremoris MG1363* | | YP_001032579 | | | Lactobacillales |
| *Lactococcus lactis subsp. cremoris SK11* | | YP_809252 | | | Lactobacillales |
| *Lactococcus lactis subsp. lactis Il1403* | | NP_267386 | | | Lactobacillales |
| *Leuconostoc mesenteroides subsp. mesenteroides ATCC 8293* | | YP_818229 | YP_817910 | | Lactobacillales |
| *Leuconostoc citreum KM20* | | YP_001728008 | YP_001728672 | | Lactobacillales |
| *Listeria innocua Clip11262* | | NP_470650 | | | Bacillales |
| *Listeria monocytogenes EGD-e* | | NP_464800 | NP_466278 | | Bacillales |
| *Listeria monocytogenes str. 4b F2365* | | YP_013891 | YP_015332 | | Bacillales |
| *Listeria welshimeri serovar 6b str. SLCC5334* | | YP_849489 | YP_850900 | | Bacillales |
| *Lysinibacillus sphaericus C3-41* | | | YP_001698117,YP_001696638 | | Bacillales |
| *Mesoplasma florum L1* | | YP_053894 | | | Mollicutes |
| *Moorella thermoacetica ATCC 39073* | | YP_429883 | YP_429226 | | Thermoanaerobacteriales |
| *Mycoplasma agalactiae PG2* | | YP_001256417 | | | Mollicutes |
| *Mycoplasma capricolum subsp. capricolum ATCC 27343* | | YP_424741 | | | Mollicutes |
| *Mycoplasma gallisepticum R* | | NP_853416 | | | Mollicutes |
| *Mycoplasma genitalium G37* | | NP_072784 | | | Mollicutes |
| *Mycoplasma hyopneumoniae 232* | | YP_115611 | | | Mollicutes |
| *Mycoplasma hyopneumoniae 7448* | | YP_287680 | | | Mollicutes |
| *Mycoplasma hyopneumoniae J* | | YP_279077 | | | Mollicutes |
| *Mycoplasma mobile 163K* | | YP_015779 | | | Mollicutes |
| *Mycoplasma mycoides subsp. mycoides SC str. PG1* | | NP_975898 | | | Mollicutes |
| *Mycoplasma penetrans HF-2* | | NP_757800 | | | Mollicutes |
| *Mycoplasma pneumoniae M129* | | NP_109949 | | | Mollicutes |
| *Mycoplasma pulmonis UAB CTIP* | | NP_326302 | | | Mollicutes |
| *Mycoplasma synoviae 53* | | YP_278650 | | | Mollicutes |
| *Oceanobacillus iheyensis HTE831* | | NP_692467 | NP_693511 | | Bacillales |
| *Oenococcus oeni PSU-1* | | YP_810595 | YP_809757 | | Lactobacillales |
| *Onion yellows phytoplasma OY-M* | | NP_950531 | | | Mollicutes |
| *Pediococcus pentosaceus ATCC 25745* | | YP_804463 | | | Lactobacillales |
| *Pelotomaculum thermopropionicum SI* | | YP_001211796 | | | Clostridiales |
| *Staphylococcus aureus RF122* | | YP_416592 | | | Bacillales |
| *Staphylococcus aureus subsp. aureus COL* | | YP_186125 | YP_187053 | | Bacillales |
| *Staphylococcus aureus subsp. aureus JH1* | | YP_001316473 | YP_001317434 | | Bacillales |
| *Staphylococcus aureus subsp. aureus JH9* | | YP_001246683 | YP_001247637 | | Bacillales |
| *Staphylococcus aureus subsp. aureus MRSA252* | | YP_040637 | YP_041694 | | Bacillales |
| *Staphylococcus aureus subsp. aureus MSSA476* | | YP_043310 | YP_044257 | | Bacillales |
| *Staphylococcus aureus subsp. aureus Mu3* | | YP_001441830 | YP_001442828 | | Bacillales |
| *Staphylococcus aureus subsp. aureus Mu50* | | NP_371774 | NP_372778 | | Bacillales |
| *Staphylococcus aureus subsp. aureus MW2* | | NP_645950 | NP_646990 | | Bacillales |
| *Staphylococcus aureus subsp. aureus N315* | | NP_374366 | NP_375367 | | Bacillales |
| *Staphylococcus aureus subsp. aureus NCTC 8325* | | YP_499758 | YP_500982 | | Bacillales |
| *Staphylococcus aureus subsp. aureus str. Newman* | | YP_001332194 | YP_001333190 | | Bacillales |
| *Staphylococcus aureus subsp. aureus USA300* | | YP_493840 | YP_494843,YP_492704 | | Bacillales |
| *Staphylococcus aureus subsp. aureus USA300_TCH1516* | | YP_001575075 | YP_001576107 | | Bacillales |
| *Staphylococcus epidermidis ATCC 12228* | | NP_764481 | NP_765383,NP_765037 | | Bacillales |
| *Staphylococcus epidermidis RP62A* | | YP_188398 | YP_189399 | | Bacillales |
| *Staphylococcus haemolyticus JCSC1435* | | YP_253579 | YP_252713 | | Bacillales |
| *Staphylococcus saprophyticus subsp. saprophyticus ATCC 15305* | | YP_301608 | YP_300749 | | Bacillales |
| *Streptococcus agalactiae 2603V/R* | | NP_688016 | | | Lactobacillales |
| *Streptococcus agalactiae A909* | | YP_329721 | | | Lactobacillales |
| *Streptococcus agalactiae NEM316* | | NP_735489 | NP_734855,NP_735167,NP_735440 | | Lactobacillales |

| | | | | | |
|---|---|---|---|---|---|
| *Streptococcus gordonii str. Challis substr. CH1* | | YP_001450484 | | | Lactobacillales |
| *Streptococcus mutans UA159* | | NP_721397 | | | Lactobacillales |
| *Streptococcus pneumoniae D39* | | YP_816589 | | | Lactobacillales |
| *Streptococcus pneumoniae Hungary19A-6* | | YP_001694689 | | | Lactobacillales |
| *Streptococcus pneumoniae R6* | | NP_358734 | | | Lactobacillales |
| *Streptococcus pneumoniae TIGR4* | | NP_345727 | | | Lactobacillales |
| *Streptococcus pyogenes M1 GAS* | | NP_269314 | YP_603190 | | Lactobacillales |
| *Streptococcus pyogenes MGAS10270* | | YP_598608 | | | Lactobacillales |
| *Streptococcus pyogenes MGAS10394* | | YP_060199 | | | Lactobacillales |
| *Streptococcus pyogenes MGAS10750* | | YP_602528 | | | Lactobacillales |
| *Streptococcus pyogenes MGAS2096* | | YP_600555 | | | Lactobacillales |
| *Streptococcus pyogenes MGAS315* | | NP_664624 | | | Lactobacillales |
| *Streptococcus pyogenes MGAS5005* | | YP_282248 | | | Lactobacillales |
| *Streptococcus pyogenes MGAS6180* | | YP_280327 | | | Lactobacillales |
| *Streptococcus pyogenes MGAS8232* | | NP_607246 | | | Lactobacillales |
| *Streptococcus pyogenes MGAS9429* | | YP_596734 | | | Lactobacillales |
| *Streptococcus pyogenes SSI-1* | | NP_802283 | | | Lactobacillales |
| *Streptococcus pyogenes str. Manfredo* | | YP_001128464 | | | Lactobacillales |
| *Streptococcus sanguinis SK36* | | YP_001035139 | | | Lactobacillales |
| *Streptococcus suis 05ZYH33* | | YP_001198351 | | | Lactobacillales |
| *Streptococcus suis 98HAH33* | | YP_001200558 | | | Lactobacillales |
| *Streptococcus thermophilus CNRZ1066* | | YP_141300 | | | Lactobacillales |
| *Streptococcus thermophilus LMD-9* | | YP_820344 | | | Lactobacillales |
| *Streptococcus thermophilus LMG 18311* | | YP_139379 | | | Lactobacillales |
| *Symbiobacterium thermophilum IAM 14863* | | YP_075310 | YP_075501 | | Lactobacillales |
| *Syntrophomonas wolfei subsp. wolfei str. Goettingen* | | YP_753530 | YP_753202 | | Clostridiales |
| *Thermoanaerobacter pseudethanolicus ATCC 33223* | | YP_001665254 | | | Thermoanaerobacteriales |
| *Thermoanaerobacter sp. X514* | | YP_001663322 | | | Thermoanaerobacteriales |
| *Thermoanaerobacter tengcongensis MB4* | | NP_623067 | | NP_623335 | Thermoanaerobacteriales |
| *Ureaplasma parvum serovar 3 str. ATCC 27815* | | YP_001752675 | | | Mollicutes |
| *Ureaplasma parvum serovar 3 str. ATCC 700970* | | NP_078429 | | | Mollicutes |
| **Fusobacteria** | | | | | |
| *Fusobacterium nucleatum subsp. nucleatum ATCC 25586* | | NP_603966 | | | Fusobacteriaceae |
| **Alphaproteobacteria** | | | | | |
| *Acidiphilium cryptum JF-5* | | YP_001233869 | | | Rhodospirillales |
| *Agrobacterium tumefaciens str. C58* | NP_356605 | NP_354310 | | | Rhizobiales |
| *Anaplasma marginale str. St. Maries* | | YP_153787 | | | Rickettsiales |
| *Anaplasma phagocytophilum HZ* | | YP_505187 | | | Rickettsiales |
| *Azorhizobium caulinodans ORS 571* | | YP_001524777 | | | Rhizobiales |
| *Bartonella bacilliformis KC583* | | YP_989131 | | | Rhizobiales |
| *Bartonella henselae str. Houston-1* | | YP_033622 | | | Rhizobiales |
| *Bartonella quintana str. Toulouse* | | YP_032289 | | | Rhizobiales |
| *Bartonella tribocorum CIP 105476* | | YP_001609502 | | | Rhizobiales |
| *Bradyrhizobium japonicum USDA 110* | NP_767212 | NP_771751 | | | Rhizobiales |
| *Bradyrhizobium sp. BTAi1* | YP_001236532 | YP_001240665 | | | Rhizobiales |
| *Bradyrhizobium sp. ORS278* | YP_001202546 | YP_001206466 | | | Rhizobiales |
| *Brucella abortus biovar 1 str. 9-941* | | YP_223392 | | | Rhizobiales |
| *Brucella canis ATCC 23365* | | YP_001594552 | | | Rhizobiales |
| *Brucella melitensis 16M* | | NP_541644 | | | Rhizobiales |
| *Brucella melitensis biovar Abortus 2308* | | YP_418811 | | | Rhizobiales |
| *Brucella ovis ATCC 25840* | | YP_001257591 | | | Rhizobiales |
| *Brucella suis 1330* | | NP_699788 | | | Rhizobiales |

| | | | | |
|---|---|---|---|---|
| *Brucella suis ATCC 23445* | | YP_001622411 | | Rhizobiales |
| *Candidatus Pelagibacter ubique HTCC1062* | | YP_266491 | | Rickettsiales |
| *Caulobacter crescentus CB15* | | NP_421254 | | Caulobacterales |
| *Caulobacter sp. K31* | YP_001685265 | YP_001683133 | | Caulobacterales |
| *Dinoroseobacter shibae DFL 12* | | YP_001532480 | | Rhodobacterales |
| *Ehrlichia canis str. Jake* | | YP_302965 | | Rickettsiales |
| *Ehrlichia chaffeensis str. Arkansas* | | YP_507548 | | Rickettsiales |
| *Ehrlichia ruminantium str. Gardel* | | YP_196269 | | Rickettsiales |
| *Ehrlichia ruminantium str. Welgevonden* | | YP_180204 | | Rickettsiales |
| *Ehrlichia ruminantium str. Welgevonden* | | YP_197223 | | Rickettsiales |
| *Erythrobacter litoralis HTCC2594* | YP_458571 | YP_457851 | | Sphingomonadales |
| *Gluconacetobacter diazotrophicus PAI 5* | | YP_001600342 | | Rhodospirillales |
| *Gluconobacter oxydans 621H* | | YP_191680 | | Rhodospirillales |
| *Granulibacter bethesdensis CGDNIH1* | | YP_744625 | | Rhodospirillales |
| *Hyphomonas neptunium ATCC 15444* | | YP_760971 | | Rhodobacterales |
| *Jannaschia sp. CCS1* | YP_508220 | YP_511102 | | Rhodobacterales |
| *Magnetospirillum magneticum AMB-1* | | YP_420045 | | Rhodospirillales |
| *Maricaulis maris MCS10* | | YP_756512 | | Rhodobacterales |
| *Mesorhizobium loti MAFF303099* | | NP_102557 | | Rhizobiales |
| *Mesorhizobium sp. BNC1* | YP_675314 | YP_673915 | | Rhizobiales |
| *Methylobacterium sp. 4-46* | YP_001771806 | YP_001771127 | YP_001770987 | Rhizobiales |
| *Methylobacterium extorquens PA1* | YP_001639444 | YP_001640766 | | Rhizobiales |
| *Methylobacterium radiotolerans JCM 2831* | YP_001753451 | YP_001753697,YP_001766487 | YP_001766643 | Rhizobiales |
| *Neorickettsia sennetsu str. Miyayama* | | YP_506474 | | Rickettsiales |
| *Nitrobacter hamburgensis X14* | YP_579129 | YP_577687 | | Rhizobiales |
| *Nitrobacter winogradskyi Nb-255* | | YP_318325 | | Rhizobiales |
| *Novosphingobium aromaticivorans DSM 12444* | YP_497864 | YP_497077 | | Sphingomonadales |
| *Ochrobactrum anthropi ATCC 49188* | YP_001372842 | YP_001372206 | | Rhizobiales |
| *Orientia tsutsugamushi Boryong* | | YP_001249094 | | Rickettsiales |
| *Paracoccus denitrificans PD1222* | | YP_914467 | | Rhodobacterales |
| *Parvibaculum lavamentivorans DS-1* | | YP_001414095 | YP_001414646 | Rhizobiales |
| *Rhizobium etli CFN 42* | YP_472371 | YP_469164 | | Rhizobiales |
| *Rhizobium leguminosarum bv. viciae 3841* | YP_771338 | YP_767339 | | Rhizobiales |
| *Rhodobacter sphaeroides 2.4.1* | YP_354029 | YP_352231 | | Rhodobacterales |
| *Rhodobacter sphaeroides ATCC 17025* | YP_001169159 | YP_001168510 | | Rhodobacterales |
| *Rhodobacter sphaeroides ATCC 17029* | YP_001044479 | YP_001042733 | | Rhodobacterales |
| *Rhodopseudomonas palustris BisA53* | | YP_782291 | | Rhizobiales |
| *Rhodopseudomonas palustris BisB18* | | YP_532112 | | Rhizobiales |
| *Rhodopseudomonas palustris BisB5* | | YP_570160 | | Rhizobiales |
| *Rhodopseudomonas palustris CGA009* | | NP_948463 | NP_949472 | Rhizobiales |
| *Rhodopseudomonas palustris HaA2* | | YP_486035 | | Rhizobiales |
| *Rhodospirillum rubrum ATCC 11170* | | YP_428284 | | Rhodospirillales |
| *Rickettsia akari str. Hartford* | | YP_001493295 | | Rickettsiales |
| *Rickettsia bellii OSU 85-389* | | YP_001496307 | | Rickettsiales |
| *Rickettsia bellii RML369-C* | | YP_537882 | | Rickettsiales |
| *Rickettsia canadensis str. McKiel* | | YP_001492485 | | Rickettsiales |
| *Rickettsia conorii str. Malish 7* | | NP_360086 | | Rickettsiales |
| *Rickettsia felis URRWXCal2* | | YP_246546 | | Rickettsiales |
| *Rickettsia massiliae MTU5* | | YP_001499231 | | Rickettsiales |
| *Rickettsia prowazekii str. Madrid E* | | NP_220709 | | Rickettsiales |
| *Rickettsia rickettsii str. Iowa* | | YP_001649818 | | Rickettsiales |
| *Rickettsia rickettsii str. 'Sheila Smith'* | | YP_001494563 | | Rickettsiales |

| | | | | |
|---|---|---|---|---|
| *Rickettsia typhi str. Wilmington* | | YP_067279 | | Rickettsiales |
| *Roseobacter denitrificans OCh 114* | | YP_682413 | | Rhodobacterales |
| *Silicibacter pomeroyi DSS-3* | | YP_168282 | | Rhodobacterales |
| *Silicibacter sp. TM1040* | | YP_614333 | | Rhodobacterales |
| *Sinorhizobium medicae WSM419* | YP_001313147 | YP_001326622 | | Rhizobiales |
| *Sinorhizobium meliloti 1021* | NP_437814 | NP_385406 | | Rhizobiales |
| *Sphingomonas wittichii RW1* | YP_001260160,YP_001263436 | YP_001264309 | | Sphingomonadales |
| *Sphingopyxis alaskensis RB2256* | | YP_616260 | | Sphingomonadales |
| *Wolbachia endosymbiont of Drosophila melanogaster* | | NP_966850 | | Rickettsiales |
| *Wolbachia endosymbiont strain TRS of Brugia malayi* | | YP_197871 | | Rickettsiales |
| *Xanthobacter autotrophicus Py2* | | YP_001418795 | | Rhizobiales |
| *Zymomonas mobilis subsp. mobilis ZM4* | | YP_162928 | | Sphingomonadales |
| **Betaproteobacteria** | | | | |
| *Acidovorax avenae subsp. citrulli AAC00-1* | YP_969856 | | YP_968937,YP_972936 | Burkholderiales |
| *Acidovorax sp. JS42* | | | YP_985731,YP_985607,YP_986473,YP_974145,YP_988170 | Burkholderiales |
| *Azoarcus sp. BH72* | YP_933299 | | YP_931600 | Rhodocyclales |
| *Azoarcus sp. EbN1* | YP_160972 | | YP_195373,YP_158408,YP_195596,YP_158693 | Rhodocyclales |
| *Bordetella bronchiseptica RB50* | NP_889376 | | NP_891494 | Burkholderiales |
| *Bordetella parapertussis 12822* | NP_884889 | | NP_886500 | Burkholderiales |
| *Bordetella pertussis Tohama I* | | | NP_879317 | Burkholderiales |
| *Bordetella petrii DSM 12804* | YP_001631006 | | YP_001630053,YP_001629640,YP_001632921,YP_001629903,YP_001628636 | Burkholderiales |
| *Burkholderia ambifaria AMMD* | YP_778070 | | YP_771691,YP_775075 | Burkholderiales |
| *burkholderia ambifaria MC40-6* | YP_001815913 | | YP_001809760 | Burkholderiales |
| *Burkholderia cenocepacia AU 1054* | YP_625473 | | YP_622389 | Burkholderiales |
| *Burkholderia cenocepacia HI2424* | YP_839621 | | YP_836771 | Burkholderiales |
| *Burkholderia cenocapacia MC0-3* | YP_001774097 | | YP_001766426 | Burkholderiales |
| *Burkholderia mallei ATCC 23344* | | | YP_101979 | Burkholderiales |
| *Burkholderia mallei NCTC 10229* | | | YP_001028234 | Burkholderiales |
| *Burkholderia mallei NCTC 10247* | | | YP_001081875 | Burkholderiales |
| *Burkholderia mallei SAVP1* | | | YP_994105 | Burkholderiales |
| *Burkholderia multivorans ATCC 17616* | YP_001585914 | | YP_001581308 | Burkholderiales |
| *Burkholderia pseudomallei 1106a* | | | YP_001064439 | Burkholderiales |
| *Burkholderia pseudomallei 1710b* | | | YP_331758 | Burkholderiales |
| *Burkholderia pseudomallei 668* | | | YP_001057200 | Burkholderiales |
| *Burkholderia pseudomallei K96243* | | | YP_106745 | Burkholderiales |
| *Burkholderia sp. 383* | | | YP_370719 | Burkholderiales |
| *Burkholderia thailandensis E264* | | | YP_440684 | Burkholderiales |
| *Burkholderia vietnamiensis G4* | YP_001115703 | | YP_001119960,YP_001121114,YP_001110078,YP_001110413,YP_001110058 | Burkholderiales |
| *Burkholderia xenovorans LB400* | YP_554144 | | YP_559782,YP_556633 | Burkholderiales |
| *Chromobacterium violaceum ATCC 12472* | | NP_903939 | | Neisseriales |
| *Dechloromonas aromatica RCB* | | | YP_283247 | Rhodocyclales |
| *Delftia acidovorans SPH-1* | YP_001565359 | | YP_001565166,YP_001561915 | Burkholderiales |
| *Herminiimonas arsenicoxydans* | | | YP_001100248,YP_001098479 | Burkholderiales |
| *Janthinobacterium sp. Marseille* | | | YP_001351829 | Burkholderiales |
| *Leptothrix cholodnii SP 6* | | | YP_001789269 | Burkholderiales |
| *Methylibium petroleiphilum PM1* | | | YP_001021533,YP_001019462,YP_001023411 | Burkholderiales |
| *Methylobacillus flagellatus KT* | YP_544669 | YP_544339 | | Methylophilales |
| *Neisseria gonorrhoeae FA 1090* | | YP_208895 | | Neisseriales |
| *Neisseria meningitidis 053442* | | YP_001600116 | | Neisseriales |
| *Neisseria meningitidis FAM18* | | YP_974241 | | Neisseriales |
| *Neisseria meningitidis MC58* | | NP_273176 | | Neisseriales |
| *Neisseria meningitidis Z2491* | | NP_283005 | | Neisseriales |

| | | | | | |
|---|---|---|---|---|---|
| *Nitrosomonas europaea ATCC 19718* | | | NP_841983 | | Nitrosomonadales |
| *Nitrosomonas eutropha C91* | | | YP_746369,YP_746639 | | Nitrosomonadales |
| *Nitrosospira multiformis ATCC 25196* | YP_410740 | | YP_411096 | | Nitrosomonadales |
| *Polaromonas naphthalenivorans CJ2* | | | YP_984179 | | Burkholderiales |
| *Polaromonas sp. JS666* | | | YP_551491 | | Burkholderiales |
| *Polynucleobacter necessarius STIR1* | | | YP_001798447 | | Burkholderiales |
| *Polynucleobacter sp. QLW-P1DMWA-1* | | | YP_001156856 | | Burkholderiales |
| *Ralstonia eutropha H16* | YP_725990 | | YP_728128 | | Burkholderiales |
| *Ralstonia eutropha JMP134* | YP_298569 | | YP_293678,YP_297614 | | Burkholderiales |
| *Ralstonia metallidurans CH34* | | | YP_584446,YP_585710 | | Burkholderiales |
| *Ralstonia solanacearum GMI1000* | | | NP_518187 | | Burkholderiales |
| *Rhodoferax ferrireducens T118* | | | YP_525185 | | Burkholderiales |
| *Thiobacillus denitrificans ATCC 25259* | YP_314503 | YP_313768 | | | Hydrogenophilales |
| *Verminephrobacter eiseniae EF01-2* | | | YP_997925 | | Burkholderiales |
| **Deltaproteobacteria** | | | | | |
| *Anaeromyxobacter dehalogenans 2CP-C* | YP_466158 | YP_465906 | | | Myxococcales |
| *Anaeromyxobacter sp. Fw109-5* | YP_001378897 | YP_001379878 | | | Myxococcales |
| *Bdellovibrio bacteriovorus HD100* | | NP_967909 | | | Bdellovibrionales |
| *Desulfococcus oleovorans Hxd3* | | YP_001530326 | | | Desulfobacterales |
| *Desulfotalea psychrophila LSv54* | | | YP_066353 | | Desulfobacterales |
| *Desulfovibrio desulfuricans G20* | | YP_386602 | YP_387814 | | Desulfovibrionales |
| *Desulfovibrio vulgaris subsp. vulgaris DP4* | | YP_965462 | YP_966391 | | Desulfovibrionales |
| *Desulfovibrio vulgaris subsp. vulgaris str. Hildenborough* | | YP_012598 | YP_011529 | | Desulfovibrionales |
| *Geobacter metallireducens GS-15* | | YP_383858 | | | Desulfuromonadales |
| *Geobacter sulfurreducens PCA* | | NP_953595 | | | Desulfuromonadales |
| *Geobacter uraniireducens Rf4* | | YP_001232415 | | | Desulfuromonadales |
| *Lawsonia intracellularis PHE/MN1-00* | | YP_595029 | | | Desulfovibrionales |
| *Myxococcus xanthus DK 1622* | | YP_631211 | YP_628804 | | Myxococcales |
| *Pelobacter carbinolicus DSM 2380* | | YP_355836 | | | Desulfuromonadales |
| *Pelobacter propionicus DSM 2379* | | YP_900217 | | | Desulfuromonadales |
| *Sorangium cellulosum 'So ce 56'* | YP_001611798 | YP_001615758 | YP_001614054 | | Myxococcales |
| *Syntrophobacter fumaroxidans MPOB* | | YP_844349 | | | Syntrophobacterales |
| *Syntrophus aciditrophicus SB* | | YP_461419 | | | Syntrophobacterales |
| **Epsilonproteobacteria** | | | | | |
| *Arcobacter butzleri RM4018* | | YP_001491118 | | | Campylobacterales |
| *Campylobacter concisus 13826* | | YP_001467625 | | | Campylobacterales |
| *Campylobacter curvus 525.92* | | YP_001408974 | | | Campylobacterales |
| *Campylobacter fetus subsp. fetus 82-40* | | YP_891380 | | | Campylobacterales |
| *Campylobacter hominis ATCC BAA-381* | | YP_001407082 | YP_001405772 | | Campylobacterales |
| *Campylobacter jejuni RM1221* | | YP_179824 | | | Campylobacterales |
| *Campylobacter jejuni subsp. doylei 269.97* | | YP_001398963 | | | Campylobacterales |
| *Campylobacter jejuni subsp. jejuni 81116* | | YP_001483157 | | | Campylobacterales |
| *Campylobacter jejuni subsp. jejuni 81-176* | | YP_001001325,YP_980071 | YP_001004049 | | Campylobacterales |
| *Campylobacter jejuni subsp. jejuni NCTC 11168* | | NP_282812 | | | Campylobacterales |
| *Helicobacter acinonychis str. Sheeba* | | YP_665198 | | | Campylobacterales |
| *Helicobacter hepaticus ATCC 51449* | | NP_861329 | | | Campylobacterales |
| *Helicobacter pylori 26695* | | NP_206916,NP_207238 | | | Campylobacterales |
| *Helicobacter pylori HPAG1* | | YP_626857 | | | Campylobacterales |
| *Helicobacter pylori J99* | | NP_222829,NP_223637,NP_223648 | | | Campylobacterales |
| *Nitratiruptor sp. SB155-2* | | YP_001357114 | | YP_001357221 | unclassified |
| *Sulfurimonas denitrificans DSM 1251* | | YP_392687 | | | Campylobacterales |
| *Sulfurovum sp. NBC37-1* | | YP_001357405 | | | unclassified |

| | | | |
|---|---|---|---|
| *Wolinella succinogenes DSM 1740* | NP_907653 | | Campylobacterales |
| **Gammaproteobacteria** | | | |
| *Acinetobacter baumannii ATCC 17978* | YP_001083496,YP_001083702 | | Pseudomonadales |
| *Acinetobacter baumannii AYE* | YP_001715103 | | Pseudomonadales |
| *Acinetobacter baumannii SDF* | YP_001708199 | | Pseudomonadales |
| *Acinetobacter sp. ADP1* | YP_045242 | | Pseudomonadales |
| *Actinobacillus pleuropneumoniae L20* | YP_001053401 | | Pasteurellales |
| *Actinobacillus pleuropneumoniae serovar 3 str. JL03* | YP_001651707 | YP_001053449,YP_001651753 | Pasteurellales |
| *Actinobacillus succinogenes 130Z* | YP_001344693 | YP_001344993 | Pasteurellales |
| *Aeromonas hydrophila subsp. hydrophila ATCC 7966* | YP_857604 | YP_858472 | Aeromonadales |
| *Aeromonas salmonicida subsp. salmonicida A449* | YP_001142853 | YP_001140206YP_001144217 | Aeromonadales |
| *Alcanivorax borkumensis SK2* | YP_692732 | | Oceanospirillales |
| *Alkalilimnicola ehrlichei MLHE-1* | YP_743462 | | Chromatiales |
| *Baumannia cicadellinicola str. Hc (Homalodisca coagulata)* | YP_588751 | | unclassified |
| *Buchnera aphidicola str. APS (Acyrthosiphon pisum)* | NP_240108 | | Enterobacteriales |
| *Buchnera aphidicola str. Bp (Baizongia pistaciae)* | | | Enterobacteriales |
| *Buchnera aphidicola str. Cc (Cinara cedri)* | | | Enterobacteriales |
| *Buchnera aphidicola str. Sg (Schizaphis graminum)* | NP_660620 | | Enterobacteriales |
| *Candidatus Blochmannia floridanus* | | | Enterobacteriales |
| *Candidatus Blochmannia pennsylvanicus str. BPEN* | | | Enterobacteriales |
| *Candidatus Carsonella ruddii PV* | | | unclassified |
| *Candidatus Ruthia magnifica str. Cm (Calyptogena magnifica)* | YP_904210 | | sulfur-oxidizing symbionts |
| *Candidatus Vesicomyosocius okutanii HA* | YP_001219759 | | sulfur-oxidizing symbionts |
| *Chromohalobacter salexigens DSM 3043* | YP_573612 | YP_574196 | Oceanospirillales |
| *Citrobacter koseri ATCC BAA-895* | YP_001452924 | YP_001453355 | Enterobacteriales |
| *Colwellia psychrerythraea 34H* | YP_267940 | YP_271431 | Alteromonadales |
| *Coxiella burnetii Dugway 5J108-111* | YP_001425405 | | Legionellales |
| *Coxiella burnetii RSA 331* | YP_001595977 | | Legionellales |
| *Coxiella burnetii RSA 493* | NP_820975 | | Legionellales |
| *Dichelobacter nodosus VCS1703A* | YP_001209082 | | Cardiobacteriales |
| *Enterobacter sakazakii ATCC BAA-894* | YP_001437661 | YP_001438246 | Enterobacteriales |
| *Enterobacter sp. 638* | YP_001176922 | YP_001176414 | Enterobacteriales |
| *Erwinia carotovora subsp. atroseptica SCRI1043* | | YP_050432,YP_048644 | Enterobacteriales |
| *Escherichia coli 536* | YP_669237 | YP_669615 | Enterobacteriales |
| *Escherichia coli DH10B* | YP_001730271 | YP_001730740 | Enterobacteriales |
| *Escherichia coli APEC O1* | YP_852447 | YP_852847 | Enterobacteriales |
| *Escherichia coli CFT073* | NP_753645 | NP_754060 | Enterobacteriales |
| *Escherichia coli E24377A* | YP_001462571 | YP_001463061 | Enterobacteriales |
| *Escherichia coli HS* | YP_001458097 | YP_001458543 | Enterobacteriales |
| *Escherichia coli K12* | NP_415790 | NP_416277 | Enterobacteriales |
| *Escherichia coli O157:H7 EDL933* | NP_287925 | NP_288196 | Enterobacteriales |
| *Escherichia coli O157:H7 str. Sakai* | NP_309873 | NP_310496 | Enterobacteriales |
| *Escherichia coli SECEC SMS-3-5* | YP_001743911 | YP_001743486 | Enterobacteriales |
| *Escherichia coli UTI89* | AP_001900 | YP_540965 | Enterobacteriales |
| *Escherichia coli W3110* | YP_540552 | AP_002382 | Enterobacteriales |
| *Francisella philomiragia subsp. Philomiragia ATCC25017* | YP_001677125 | | Thiotrichales |
| *Francisella tularensis subsp. holarctica* | YP_001427881 | | Thiotrichales |
| *Francisella tularensis subsp. holarctica FTA* | YP_763048 | | Thiotrichales |
| *Francisella tularensis subsp. holarctica OSU18* | YP_513198 | | Thiotrichales |
| *Francisella tularensis subsp. novicida U112* | YP_898089 | | Thiotrichales |
| *Francisella tularensis subsp. tularensis FSC198* | YP_667035 | | Thiotrichales |
| *Francisella tularensis subsp. tularensis SCHU S4* | YP_169903 | | Thiotrichales |

| | | | | |
|---|---|---|---|---|
| *Francisella tularensis subsp. tularensis WY96-3418* | | YP_001122172 | | Thiotrichales |
| *Haemophilus ducreyi 35000HP* | | NP_873809 | NP_873711,NP_873446 | Pasteurellales |
| *Haemophilus influenzae 86-028NP* | | YP_249232 | YP_248156,YP_247759 | Pasteurellales |
| *Haemophilus influenzae PittEE* | | YP_001290656 | YP_001290029 | Pasteurellales |
| *Haemophilus influenzae PittGG* | | YP_001291588 | YP_001292393 | Pasteurellales |
| *Haemophilus influenzae Rd KW20* | | NP_439516 | NP_438605 | Pasteurellales |
| *Haemophilus somnus 129PT* | | YP_719124 | YP_719522,YP_719942 | Pasteurellales |
| *Haemophilus somnus 2336* | | YP_001784712 | YP_001783658,YP_001785040 | Pasteurellales |
| *Hahella chejuensis KCTC 2396* | | YP_435874 | | Oceanospirillales |
| *Halorhodospira halophila SL1* | | YP_001003891 | | Chromatiales |
| *Idiomarina loihiensis L2TR* | | YP_155399 | | Alteromonadales |
| *Klebsiella pneumoniae subsp. pneumoniae MGH 78578* | | YP_001334933 | YP_001334871 | Enterobacteriales |
| *Legionella pneumophila str. Corby* | | YP_001249874 | | Legionellales |
| *Legionella pneumophila str. Lens* | | YP_127851 | | Legionellales |
| *Legionella pneumophila str. Paris* | | YP_124957 | | Legionellales |
| *Legionella pneumophila subsp. pneumophila str. Philadelphia 1* | | YP_096604 | | Legionellales |
| *Mannheimia succiniciproducens MBEL55E* | | YP_088288 | YP_087922 | Pasteurellales |
| *Marinobacter aquaeolei VT8* | | YP_956966,YP_958425 | YP_959791 | Alteromonadales |
| *Marinomonas sp. MWYL1* | | YP_001340971 | YP_001342115 | Oceanospirillales |
| *Methylococcus capsulatus str. Bath* | | YP_115233 | | Methylococcales |
| *Nitrosococcus oceani ATCC 19707* | | YP_344982 | | Chromatiales |
| *Pasteurella multocida subsp. multocida str. Pm70* | | NP_245552 | NP_245144 | Pasteurellales |
| *Pectobacterium atrosepticum SCRI1043* | | YP_050381 | | Enterobacteriales |
| *Photobacterium profundum SS9* | | YP_130661 | YP_130771 | Vibrionales |
| *Photorhabdus luminescens subsp. laumondii TTO1* | | NP_929674 | NP_929786,NP_928366 | Enterobacteriales |
| *Pseudoalteromonas atlantica T6c* | YP_662415 | YP_661926 | YP_660358 | Alteromonadales |
| *Pseudoalteromonas haloplanktis TAC125* | | YP_339866 | YP_340847,YP_340837 | Alteromonadales |
| *Pseudomonas aeruginosa PA7* | YP_001348358 | YP_001347520,YP_001349815 | | Pseudomonadales |
| *Pseudomonas aeruginosa PAO1* | NP_250934 | NP_251701 | | Pseudomonadales |
| *Pseudomonas aeruginosa UCBPP-PA14* | YP_790992 | YP_790161,YP_792914 | | Pseudomonadales |
| *Pseudomonas entomophila L48* | YP_607900 | YP_609232 | YP_607819 | Pseudomonadales |
| *Pseudomonas fluorescens Pf-5* | YP_260520 | YP_259061,YP_261755 | | Pseudomonadales |
| *Pseudomonas fluorescens PfO-1* | YP_348675 | YP_349604 | YP_349093,YP_348730 | Pseudomonadales |
| *Pseudomonas mendocina ymp* | YP_001188262 | YP_001187079 | | Pseudomonadales |
| *Pseudomonas putida F1* | YP_001267266 | YP_001268911 | YP_001267151,YP_001268236 | Pseudomonadales |
| *Pseudomonas putida W619* | YP_001750059 | YP_001748529 | YP_001750276,YP_001750149 | Pseudomonadales |
| *Pseudomonas putida GB-1* | | YP_001667918 | YP_001669851 | Pseudomonadales |
| *Pseudomonas putida KT2440* | NP_745961 | NP_744288 | NP_746149 | Pseudomonadales |
| *Pseudomonas stutzeri A1501* | YP_001172718 | YP_001172249 | YP_001173401 | Pseudomonadales |
| *Pseudomonas syringae pv. phaseolicola 1448A* | YP_274601 | YP_275367 | YP_272298,YP_275124 | Pseudomonadales |
| *Pseudomonas syringae pv. syringae B728a* | YP_235836 | YP_236357 | YP_235325,YP_234561 | Pseudomonadales |
| *Pseudomonas syringae pv. tomato str. DC3000* | NP_792773 | NP_793294 | NP_792290 | Pseudomonadales |
| *Psychrobacter arcticus 273-4* | | YP_264942 | | Pseudomonadales |
| *Psychrobacter cryohalolentis K5* | YP_580104 | YP_581162 | | Pseudomonadales |
| *Psychrobacter sp. PRwf-1* | YP_001279766 | YP_001279298 | | Pseudomonadales |
| *Psychromonas ingrahamii 37* | | YP_943338 | YP_943791 | Alteromonadales |
| *Saccharophagus degradans 2-40* | | YP_527255 | YP_527016 | Alteromonadales |
| *Salmonella enterica subsp. arizonae serovar 62* | | YP_001570290 | YP_001570714 | Enterobacteriales |
| *Salmonella enterica subsp. enterica serovar Choleraesuis str. SC-B67* | | YP_216698 | YP_216307 | Enterobacteriales |
| *Salmonella enterica subsp. enterica serovar Paratyphi A str. ATCC 9150* | | YP_150435 | YP_150791 | Enterobacteriales |
| *Salmonella enterica subsp. enterica serovar Paratyphi B str. SPB7* | | YP_001587763 | YP_001591453,YP_001588265 | Enterobacteriales |
| *Salmonella enterica subsp. enterica serovar Typhi str. CT18* | | NP_455783 | NP_458624,NP_456214 | Enterobacteriales |

| | | | | | |
|---|---|---|---|---|---|
| *Salmonella enterica subsp. enterica serovar Typhi str. Ty2* | | NP_805405 | NP_807836,NP_804985 | | Enterobacteriales |
| *Salmonella typhimurium LT2* | | NP_460673 | NP_460264 | | Enterobacteriales |
| *Serratia proteamaculans 568* | | YP_001478886 | YP_001478947,YP_001480330,YP_001476548 | | Enterobacteriales |
| *Shewanella amazonensis SB2B* | | YP_927199 | YP_928020 | | Alteromonadales |
| *Shewanella baltica OS155* | | YP_001050858 | YP_001051092,YP_001041729 | | Alteromonadales |
| *Shewanella baltica OS185* | | YP_001366689 | YP_001366950 | | Alteromonadales |
| *Shewanella baltica OS195* | | YP_001555037 | YP_001555256,YP_001557130 | | Alteromonadales |
| *Shewanella denitrificans OS217* | | YP_563331 | YP_563403 | | Alteromonadales |
| *Shewanella frigidimarina NCIMB 400* | | YP_751161 | YP_750091 | | Alteromonadales |
| *Shewanella halifaxensis HAW EB4* | | YP_001673830 | YP_001674850 | | Alteromonadales |
| *Shewanella loihica PV-4* | | YP_001094349 | YP_001094424 | | Alteromonadales |
| *Shewanella oneidensis MR-1* | | NP_718289 | NP_718626 | | Alteromonadales |
| *Shewanella pealeana ATCC 700345* | | YP_001501395 | YP_001501485 | | Alteromonadales |
| *Shewanella putrefaciens CN-32* | | YP_001183770 | YP_001183955 | | Alteromonadales |
| *Shewanella sediminis HAW-EB3* | | YP_001474576 | YP_001473397 | | Alteromonadales |
| *Shewanella sp. ANA-3* | | YP_869368 | YP_869126 | | Alteromonadales |
| *Shewanella sp. MR-4* | | YP_733719 | YP_733568 | | Alteromonadales |
| *Shewanella sp. MR-7* | | YP_737712 | YP_737554 | | Alteromonadales |
| *Shewanella sp. W3-18-1* | | YP_963149 | YP_962964 | | Alteromonadales |
| *Shewanella woodyi ATCC 51908* | | YP_001760182 | YP_001761342 | | Alteromonadales |
| *Shigella boydii Sb227* | | YP_408219 | YP_407779 | | Enterobacteriales |
| *Shigella dysenteriae Sd197* | | YP_402982 | YP_403139 | | Enterobacteriales |
| *Shigella flexneri 2a str. 2457T* | | NP_836967 | NP_837145 | | Enterobacteriales |
| *Shigella flexneri 2a str. 301* | | NP_707181 | NP_707350 | | Enterobacteriales |
| *Shigella flexneri 5 str. 8401* | | YP_688795 | YP_688950 | | Enterobacteriales |
| *Shigella sonnei Ss046* | | YP_310776 | YP_310338 | | Enterobacteriales |
| *Sodalis glossinidius str. 'morsitans'* | | YP_455088 | YP_455041 | | Enterobacteriales |
| *Thiomicrospira crunogena XCL-2* | | YP_390466 | | | Thiotrichales |
| *Vibrio cholerae O1 biovar eltor str. N16961* | | NP_231366 | NP_231677 | | Vibrionales |
| *Vibrio cholerae O395* | | YP_001217276 | YP_001217569 | | Vibrionales |
| *Vibrio fischeri ES114* | | YP_204434 | YP_207176,YP_205024 | | Vibrionales |
| *Vibrio harveyi ATCC BAA-1116* | | YP_001444771 | YP_001436068,YP_001446218 | | Vibrionales |
| *Vibrio parahaemolyticus RIMD 2210633* | | NP_797401 | NP_798528 | | Vibrionales |
| *Vibrio vulnificus CMCP6* | | NP_760989 | NP_761924 | | Vibrionales |
| *Vibrio vulnificus YJ016* | | NP_935107 | NP_933948 | | Vibrionales |
| *Wigglesworthia glossinidia endosymbiont of Glossina brevipalpis* | | | | | Enterobacteriales |
| *Xanthomonas axonopodis pv. citri str. 306* | NP_640391 | NP_644113 | NP_642529 | | Xanthomonadales |
| *Xanthomonas campestris pv. campestris str. 8004* | YP_241148 | YP_244885 | YP_243144 | | Xanthomonadales |
| *Xanthomonas campestris pv. campestris str. ATCC 33913* | NP_635429 | NP_639100 | | | Xanthomonadales |
| *Xanthomonas campestris pv. vesicatoria str. 85-10* | YP_361770 | YP_365663 | YP_364018,YP_364137,YP_361711 | | Xanthomonadales |
| *Xanthomonas oryzae pv. oryzae KACC10331* | YP_198814 | YP_199218 | | | Xanthomonadales |
| *Xanthomonas oryzae pv. oryzae MAFF 311018* | YP_449184 | YP_449569 | | | Xanthomonadales |
| *Xylella fastidiosa 9a5c* | | NP_061659,NP_298210 | NP_299064,NP_299339 | | Xanthomonadales |
| *Xylella fastidiosa M12* | | YP_001776438 | YP_001775704 | | Xanthomonadales |
| *Xylella fastidiosa Temecula1* | | NP_779952 | | | Xanthomonadales |
| *Yersinia enterocolitica subsp. enterocolitica 8081* | | YP_001006438 | YP_001006485 | | Enterobacteriales |
| *Yersinia pestis Angola* | | YP_001606739 | YP_001606772 | | Enterobacteriales |
| *Yersinia pestis Antiqua* | | YP_651488 | YP_651434 | | Enterobacteriales |
| *Yersinia pestis biovar Microtus str. 91001* | | NP_993355 | NP_993305 | | Enterobacteriales |
| *Yersinia pestis CO92* | | NP_405760 | NP_405710 | | Enterobacteriales |
| *Yersinia pestis KIM* | | NP_669373 | NP_669467 | | Enterobacteriales |
| *Yersinia pestis Nepal516* | | YP_647615 | YP_647561 | | Enterobacteriales |

| | | | | | |
|---|---|---|---|---|---|
| *Yersinia pestis Pestoides F* | | YP_001162290 | YP_001162342 | | Enterobacteriales |
| *Yersinia pseudotuberculosis IP 31758* | | YP_001400898 | YP_001400952,YP_001393284,YP_001393331 | | Enterobacteriales |
| *Yersinia pseudotuberculosis YPIII* | | YP_001720773 | YP_001720827 | | Enterobacteriales |
| *Yersinia pseudotuberculosis IP 32953* | | YP_070657 | YP_068538,YP_070608 | | Enterobacteriales |
| **Proteobacteria** | | | | | |
| *Magnetococcus sp. MC-1* | | YP_867551 | YP_866447 | | unclassified |
| **Spirochaetes** | | | | | |
| *Borrelia afzelii PKo* | | YP_710281 | | | Spirochaetaceae |
| *Borrelia burgdorferi B31* | | NP_212962 | | | Spirochaetaceae |
| *Borrelia garinii PBi* | | YP_073267 | | | Spirochaetaceae |
| *Leptospira borgpetersenii serovar Hardjo-bovis JB197* | | YP_800587 | | | Leptospiraceae |
| *Leptospira borgpetersenii serovar Hardjo-bovis L550* | | YP_797709 | | | Leptospiraceae |
| *Leptospira interrogans serovar Copenhageni str. Fiocruz L1-130* | | YP_002014 | | | Leptospiraceae |
| *Leptospira interrogans serovar Lai str. 56601* | | NP_711902 | | | Leptospiraceae |
| *Treponema denticola ATCC 35405* | | NP_971815 | | | Spirochaetaceae |
| *Treponema pallidum subsp. pallidum str. Nichols* | | NP_218834 | | | Spirochaetaceae |
| **Thermotogae** | | | | | |
| *Fervidobacterium nodosum Rt17-B1* | | YP_001410533 | | YP_001410867 | Thermotogaceae |
| *Petrotoga mobilis SJ95* | | YP_001568661 | | | Thermotogaceae |
| *Thermosipho melanesiensis BI429* | | YP_001306270 | | | Thermotogaceae |
| *Thermotoga lettingae TMO* | | YP_001469829 | | | Thermotogaceae |
| *Thermotoga maritima MSB8* | | NP_228071 | | NP_227988 | Thermotogaceae |
| *Thermotoga petrophila RKU-1* | | YP_001244262 | | YP_001244347 | Thermotogaceae |
